# Mapping the malaria parasite druggable genome by using in vitro evolution and chemogenomics

**DOI:** 10.1126/science.aan4472

**Published:** 2018-01-12

**Authors:** Annie N. Cowell, Eva S. Istvan, Amanda K. Lukens, Maria G. Gomez-Lorenzo, Manu Vanaerschot, Tomoyo Sakata-Kato, Erika L. Flannery, Pamela Magistrado, Edward Owen, Matthew Abraham, Gregory LaMonte, Heather J. Painter, Roy M. Williams, Virginia Franco, Maria Linares, Ignacio Arriaga, Selina Bopp, Victoria C. Corey, Nina F. Gnädig, Olivia Coburn-Flynn, Christin Reimer, Purva Gupta, James M. Murithi, Pedro A. Moura, Olivia Fuchs, Erika Sasaki, Sang W. Kim, Christine H. Teng, Lawrence T. Wang, Aslı Akidil, Sophie Adjalley, Paul A. Willis, Dionicio Siege, Olga Tanaseichuk, Yang Zhong, Yingyao Zhou, Manuel Llinás, Sabine Ottilie, Francisco-Javier Gamo, Marcus C. S. Lee, Daniel E. Goldberg, David A. Fidock, Dyann F. Wirth, Elizabeth A. Winzeler

**Affiliations:** 1 School of Medicine, University of California San Diego (UCSD), 9500 Gilman Drive, La Jolla, CA 92093, USA; 2 Departments of Medicine and Molecular Microbiology, Washington University School of Medicine, St. Louis, MO 63110, USA; 3 Department of Immunology and Infectious Disease, Harvard T.H. Chan School of Public Health, 665 Huntington Avenue, Boston, MA 02115, USA; 4 Infectious Disease Program, The Broad Institute, 415 Main Street, Cambridge, MA 02142, USA; 5 Tres Cantos Medicines Development Campus, Malaria Discovery Performance Unit, GlaxoSmithKline, Severo Ochoa 2, Tres Cantos 28760, Madrid, Spain; 6 Department of Microbiology and Immunology, Columbia University College of Physicians and Surgeons, New York, NY 10032, USA; 7 Department of Biochemistry and Molecular Biology, Pennsylvania State University, University Park, PA 16802, USA; 8 Malaria Programme, Wellcome Sanger Institute, Wellcome Genome Campus, Hinxton, Cambridgeshire CB10 1SA, UK; 9 Medicines for Malaria Venture, Post Office Box 1826, 20 Route de Pre-Bois, 1215 Geneva 15, Switzerland; 10 Skaggs School of Pharmacy and Pharmaceutical Sciences, UCSD, 9500 Gilman Drive, La Jolla, CA 92093, USA; 11 Genomics Institute of the Novartis Research Foundation, 10675 John J Hopkins Drive, San Diego, CA 92121, USA; 12 Division of Infectious Diseases, Columbia University College of Physicians and Surgeons, New York, NY 10032, USA

## Abstract

Chemogenetic characterization through in vitro evolution combined with whole-genome analysis can identify antimalarial drug targets and drug-resistance genes.We performed a genome analysis of 262 *Plasmodium falciparum* parasites resistant to 37 diverse compounds.We found 159 gene amplifications and 148 nonsynonymous changes in 83 genes associated with drug-resistance acquisition, where gene amplifications contributed to one-third of resistance acquisition events. Beyond confirming previously identified multidrug-resistance mechanisms, we discovered hitherto unrecognized drug target–inhibitor pairs, including thymidylate synthase and a benzoquinazolinone, farnesyltransferase and a pyrimidinedione, and a dipeptidylpeptidase and an arylurea.This exploration of the *P. falciparum* resistome and druggable genome will likely guide drug discovery and structural biology efforts, while also advancing our understanding of resistance mechanisms available to the malaria parasite.

Malaria has a disproportionately negative impact on human health because its causal protozoan parasites are adept at changing their genomes to evade antimalarial drugs and the human immune system. A single human infection may result in upwards of 10^12^ asexual blood-stage parasites. Thus, even with a relatively slow random mutation rate (~10^−9^ per nucleotide site per mitotic division) in the parasite, within a few cycles of replication, each base in the *P. falciparum* genome can acquire a random genetic change that may render at least one parasite resistant to the activity of a drug or a human-encoded antibody. The recent evolution of artemisinin-resistant parasites in Southeast Asia now threatens both life-saving treatments and malaria-control efforts (*1*).

Although this rapid evolution impedes our ability to control the disease, in vitro evolution in the presence of known antimalarials, followed by whole-genome sequencing of resistant clones, can be used to discover mediators of drug resistance (*2*). Testing for the evolution of resistance can also reveal antimalarial drug targets (*3*). Comparedwith targets that are validated using genetic knockdown methods, chemically validated drug targets are more valuable because their activity can be inhibited in cultured parasites by a small molecule. Furthermore, the inhibitor provides a tool for crystallization and chemical genetic studies. Most studies using this method to date have focused on single gene mutations in response to single compounds, even though, in many cases, additional allelic changes have been noted in *P. falciparum* clones during the acquisition of compound resistance.

## Next-generation sequencing shows both neutral and positive selection

We systematically studied patterns of *P. falciparum* genome evolution by analyzing the sequences of clones resistant to diverse compounds with antimalarial activity across the P. falciparum life cycle. To investigate the genomic evolutionary response to treatment with small molecules, we assembled a collection of isogenic *P. falciparum* clones that had acquired resistance to an array of chemically distinct small-molecule growth inhibitors. Of the 37 different small molecules used during in vitro resistance evolution (table S1), 26 compounds were identified as having potent in vitro antimalarial activity from previous P. falciparum phenotypic screens (*4*–*7*). Others were chosen on the basis of medicinal chemistry optimization: the spiroindoloneNITD678 (*8*) and the imidazolopiperazines GNF179, GNF452, and GNF707 (*9*). Respectively, NITD678 and the three imidazolopiperazines are similar to KAE609 and KAF156, which are recently discovered antimalarials that are nowin clinical trials (*10*, *11*).Wealso included the licensed antimalarials atovaquone and primaquine. Although three carbazoles (MMV019017, MMV009063, and MMV665882) and the three imidazolopiperazines (GNF707, GNF452, and GNF179) were structurally similar to one another (ChemAxon fingerprint score ≥ 0.7) (fig. S1), most compounds tested had a distinct variety of functional groups and heterocyclic substructures, with some compounds showing activity affecting different aspects of the parasite life cycle (tables S1 and S2 and fig. S1).

Although some *P. falciparum* clones included herein have been previously described with respect to their drug sensitivity (*12*), other clones, including those resistant to primaquine and those resistant to GNF179, were generated in this study over a period of 3 to 6 months (fig. S2) using a stepwise, high-pressure intermittent, or constant method of compound exposure (table S1). Parasite clones exhibited a gain of resistance relative to their isogenic parent clones, with a fold shift of 2.8 and 37.2 in the lower and upper quartile, respectively, of the half-maximal effective concentration (EC_50_) for resistant clones (table S3). This resistance shift remained stable after compound pressure was removed for 30 to 115 days. Genomic DNA was available from an average of 6.45 (median = 5) independently derived clones for each compound.

**Fig. 1:**
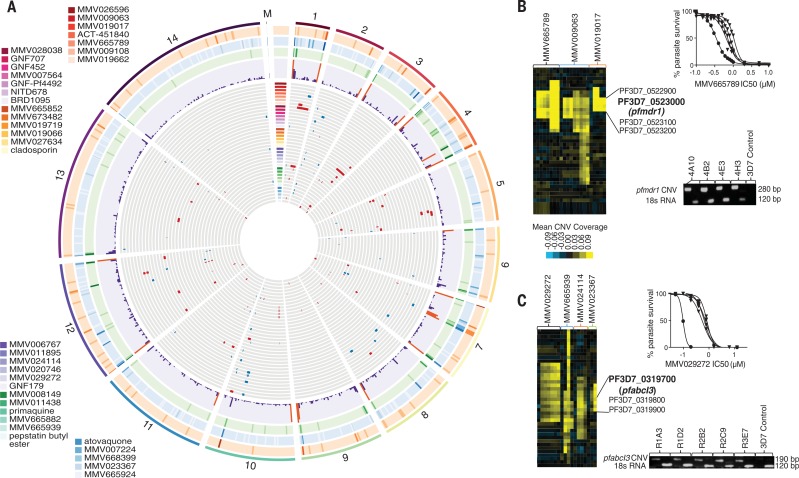
**Genetic changes acquired by 235 compound-resistant P. falciparum clones. (A)** Circos plot (*68*) summarizing single nucleotide variants (SNVs), insertions and deletions (indels), and copy number variants (CNVs) acquired by the 235 P. falciparum clones resistant to 37 diverse compounds with antimalarial activity, grouped by chromosome. Each bar on the outer three rings represents 30,000 bp, and the darkness of the bar indicates a greater number ofmutations.Orange ring,mutations lead to loss of function; blue ring, mutations lead to protein modification (nonsynonymous change or inframe deletion); green ring, no protein change (synonymous mutations or introns). The purple ring displays a histogram showing total counts of all three types of mutations, with orange bars where counts exceed 15.The gray rings display variants for resistant clones grouped by compound,with each ring representing one of 37 compounds. Red bars represent the location of CNVs, and blue bars represent the location of SNVs. **(B)** CNVs in the known drug-resistance gene pfmdr1.The heatmap (left) shows clones grouped by compound and is brighter yellowfor genes with higher coverage (>2.5-fold above the standard deviation). Resistance shifts (top right) relative to the sensitive parent (circles) were demonstrated in MMV665789-resistant clones (triangles). PCR products (bottom right) shows that resistant clones contain the pfmdr1 CNV junction sequence,whereas a 3D7 control lacks this sequence. **(C)** CNVs in pfabcI3 are associated with resistance to multiple compounds. Resistance shifts relative to the sensitive parent (circles) were demonstrated in MMV029272-resistant clones (triangles). Again, the CNV junction sequence was not found in the 3D7 control DNA, whereas it was identified in clones resistant to MMV029272.

To identify the genetic basis of drug resistance, 204 clones (including resistant clones and sensitive isogenic parent clones) were fully sequenced with the paired-end read method. We included 58 published genomic sequences of clones resistant to 12 compounds, including cladosporin (*13*), atovaquone (*14*), and GNF179 (*15*). Alignment to the *P. falciparum* reference genome showed 80.3-fold average coverage of the 23.3–million base pair (Mbp) genome, with 85.3% of bases covered by 20 or more reads (table S3). We discovered 1277 single nucleotide variants (SNVs) and 668 small insertions or deletions (indels) that arose in the 262 whole-genome sequences during resistance acquisition (Fig. 1A, Table 1, and table S4).

**Table 1:** Summary of effects for resistant clones in the core versus noncore regions of the *P. falciparum genome*. Noncore regions, making up 6% of the total genome, were located in subtelomeric regions and internal var gene clusters and encoded mostly PfEMP1s, RIFINs, and STEVORs. Effect changes are as classified using SnpEff (*67*).

Effect	Number		
	Total	Core	Noncore
**Indels**			
Codon change plus codon insertion	1	1	0
Disruptive inframe deletion	22	17	5
Disruptive inframe insertion	20	11	9
Frameshift variant	64	26	38
Frameshift variant plus stop-gained	1	0	1
Inframe deletion	43	37	6
Inframe insertion	30	20	10
Intergenic	412	239	145
Intron variant	69	64	5
Noncoding exon variant	1	1	0
Splice region variant plus intron variant	5	5	0
Total indels	668	449	219
**SNVs**			
Intergenic	700	178	522
Intron variant	49	30	19
Nonsynonymous coding	363	148	215
Splice region variant plus intron variant	3	2	1
Start lost	1	1	0
Stop-gained	10	9	1
Synonymous coding	1	0	1
Synonymous stop	1	0	1
Synonymous variant	149	21	128
Total SNVs	1277	389	888
**Total indels and SNVs**	**1945**	**838**	**1107**

Previous whole-genome analyses of *P. falciparum* with microarrays and sequencing (*14*, *16*) showed that during long-term in vitro growth, genes encoding proteins involved in antigenic variation (such as *var* and *rifin*), usually located in the subtelomeric regions, rapidly acquiremutations. Thus, we hypothesized that we would find a similar pattern for small variants in our data set. Indeed, upon verification, 1107 of these variants were located in the 6% of the genome that encodes proteins involved in antigenic variation, referred to as the noncore genome (Table 1). We further predicted that if mutations in the core genome conferred a selective advantage, then we would find an enrichment of nonsynonymous coding changes. As predicted, the ratio of nonsynonymous coding to synonymous coding alleles in the 23-Mbp core genome was 7:1 (148:21), indicating an uneven distribution (accumulative binomial P = 7.7 × 10^–10^) and detectable positive selection. In contrast, this ratio was ~2:1 (216:128) in the noncore region, indicating near-neutral selection and confirming the mutability of these genes in the absence of selection. In total, we observed 148 nonsynonymous changes (table S5) in 83 genes (table S6) in the core genome (containing 838 variants total), with less than one nonsynonymous change per resistant clone (table S7).

We also predicted that these 83 genes would be enriched for annotated roles in drug resistance, with mutations detected in the appropriate compound-specific groups of clones. Gene ontology enrichment testing revealed that the set of 83 genes was statistically enriched with a “response to drug” gene ontology biological process designation (GO:0042493; accumulative hypergeometric *P* = 5.78 × 10^–7^; table S8). To the best of our knowledge, there were no false negatives, and our results were in agreement with other studies (*8*, *17*).

## Most drug-resistant clones acquire copy number variants in addition to SNVs

Copy number variants (CNVs) also contribute to drug resistance in *P. falciparum* (*18*–*20*). For example, amplification of the gene encoding multidrugresistance protein 1 (*pfmdr1*; PF3D7_0523000) confersmultidrug resistance, including resistance to mefloquine (*21*). However, traditional methods of CNV detection based on sequencing read depth are more problematic in *P. falciparum* owing to its AT-rich content (~81%), which leads to uneven sequencing coverage (*22*). Thus, we established an automated pipeline for CNV detection using >3.0-fold normalized coverage. Briefly, average read depth was computed for coding regions, because intergenic regions of *P. falciparum* exhibit reduced alignment confidence owing to 90 to 95% AT content and AT-repeat segments (*23*). The read coverage data were normalized in three groups representing the genetic backgrounds 3D7, Dd2, or 7G8, allowing for the identification of increased read coverage in background-specific amplified regions. Sets of two or more contiguous genes showing a ~2.0-fold change relative to the mean were identified. Potential amplified regions were then filtered to yield 159 high-confidence CNVs (core genome, mean average coverage per gene set > ~3.0-fold relative to mean coverage, and Benjamini-Hochberg–corrected *P*<0.001) (tables S8 and S10). Altogether, 159 CNVs were observed in *P. falciparum* clones resistant to 27 of the 37 compounds (tables S8 and S11). CNVs were found primarily on chromosomes 1, 3, 5, and 12, with an average size of ~65 kilo–base pairs (kbp) (Fig. 1 and table S8). Of note, 76 core genome deletions were also identified (table S12), but most of these were small (<5 genes), located in subtelomeric regions, and apparently unrelated to the acquisition of drug resistance.

To validate our CNV-detection algorithm, we analyzed randomized, permuted average read coverage data and identified only about eight CNVs fulfilling these criteria. To assess our false negative rate, we confirmed that we could detect known CNVs previously detected by microarray analysis, including those that span the gene encoding lysyl tRNA synthetase ( *pf krs1*; PF3D7_1350100) in three cladosporin-resistant clones (*13*) (Benjamini-Hochberg–corrected *t* test; *P* = 1.9 × 10^–40^, 2.3 × 10^–23^, and 1.3 × 10^–12^, respectively) and the amplification event encompassing the multidrug-resistance protein 1 locus (*pfmrp1*; PF3D7_0112200) in the atovaquoneresistant clone R5a (*14*) (Benjamini-Hochberg– corrected t test; *P* = 4.1 × 10^–33^). The known amplification surrounding *pfatp4* in NITD678- resistant clones (*8*) was not detected, likely because this clone was sequenced with an older, 60-bpread- length technology and was therefore not comparable to the rest of the set (although the amplification could be visually detected when compared with other samples sequenced with 60-bp reads). All 159 CNVs could be visually identified on a heatmap of normalized sequencing coverage. We found that CNV read coverage correlated with the shift in EC_50_ in resistant clones when no other resistance-conferring mutations were present, such as the *pfmdr1* amplification in clones resistant to compound MMV665789 (Fig. 1B). Weused quantitative polymerase chain reaction (qPCR) to further validate CNVs that spanned genes encoding phosphatidylinositol 4-kinase (*pfpi4k*; PF3D7_0509800), ABC [adenosine triphosphate (ATP)–binding cassette] transporter I familymember 1 (*pfabcI3*;PF3D7_0319700), and the aminophospholipid-transporting P-ATPase (*pfatp2*; PF3D7_1219600) (fig. S3). In addition, we identified paired-end reads from sequencing library fragments that spanned the CNV boundaries in the Integrative Genomics Viewer (*24*, *25*); this was followed by PCR over the boundaries (Fig. 1, B and C, and fig. S4) (*26*) for CNVs that covered *pfmdr1*, *pfabcI3*, and *pfpi4k*.

## Resistance mechanisms are reproducible and structure-dependent

**Fig. 2:**
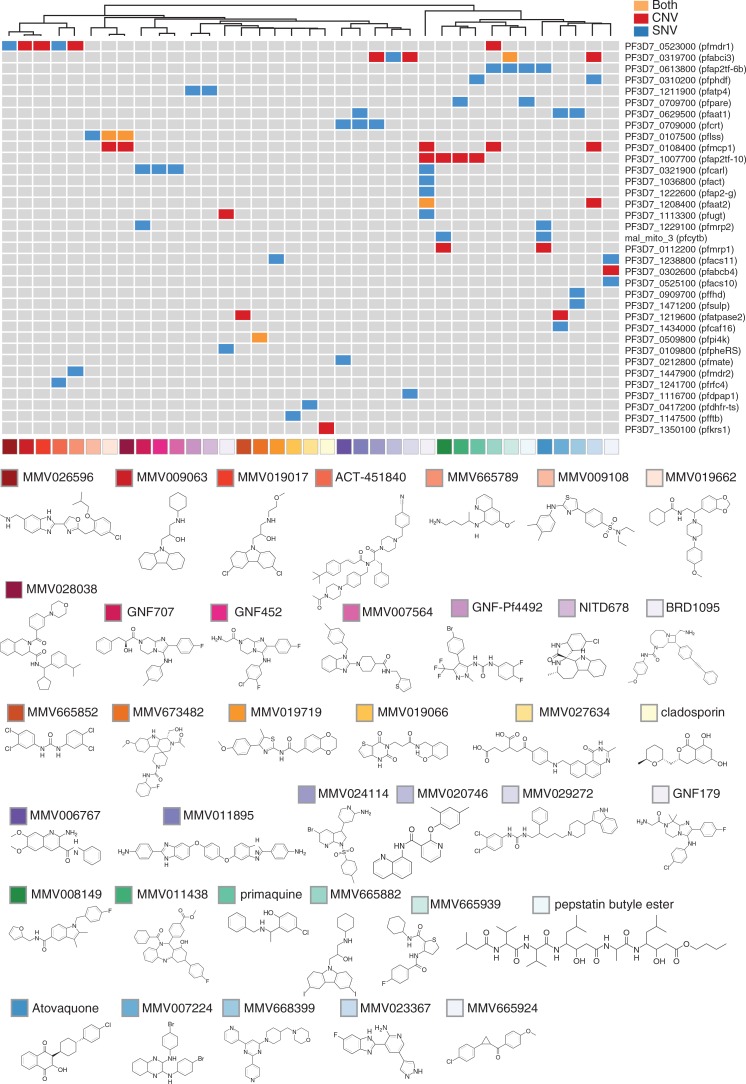
**The P. falciparum resistome.** Compounds were clustered first on the basis of their target profile similarities, then by their chemical structural similarities. Each compound was assigned a color code, which is shared with Fig. 1. Genes that were detected as mutated (CNV, SNV, or indel) in independently created clones are listed.

We predicted that treating parasites with compounds that have similar chemical structures would yield reproducible genomic changes. Clustering on the basis of compound similarity showed that parasites acquired similar resistance alleles when treatedwith structurally similar compounds (Fig. 2 and fig. S1). For example, independent clones resistant to imidazolopiperazines (GNF452, GNF707, and GNF179) and the closely related compoundMMV007564 acquired mutations resulting in coding changes in the cyclic amine resistance locus (*pfcarl*; PF3D7_0321900). Furthermore, amplification of the *pfmdr1* region was observed in all 3D7 clones that acquired resistance to three closely related carbazoles: MMV009063, MMV019017, and MMV665882 (table S10 and Fig. 2).

## The *P. falciparum* resistome

Our data set of CNVs and SNVs revealed 35 genes with two or more types of evidence suggesting a role as a drug-resistance determinant or actual target (Fig. 2). Evidence types include SNVs within an amplification event, the presence of two different alleles in the same gene for the same compound, or the same allele appearing in response to two different compounds. Mutations in *pfmdr1* were discovered for six different compounds, confirming its known role in drug resistance. Notably, a likely resistance mechanism or target gene was discovered for each compound examined. Below, we highlight genes with the strongest evidence of mediating drug resistance.

## Discovery of alleles in known drug-resistance genes

Genetic detection of alleles associated with antimalarial resistance is the primary method of tracking resistance in clinical samples, because in vitro phenotypic susceptibility testing is difficult and costly in malaria parasites. Analysis of the mutations present in our set of resistant *P. falciparum* clones revealed both known and unknown genes implicated in drug resistance. We found several previously unidentified mutations in known *P. falciparum* resistance mediators. For example, we found four hitherto unrecognized alleles in the gene encoding the *P. falciparum* chloroquine resistance transporter (*pfcrt*; PF3D7_0709000), including S65R, A138V, K76Q, and S90N amino acid changes. Although one of the selection compounds, MMV006767, bears an aminoquinoline core, similar to chloroquine, neither MMV011895 norMMV024114 do, providing evidence that *PfCRT* is a pleotropic transporter regulating drug levels in the digestive vacuole. We also discovered a F806L allele in *pfmdr1* inMMV026596-resistant lines, which showed a 1.7-fold increase in the MMV026596 EC_50_ (table S3). An equivalent increase in the MMV026596 EC_50_ was also observed (table S13) upon assaying a separate NF54 line that had been selected using an unrelated compound (ACT-451840) and that had also acquired a F806L mutation in *pfmdr1* (*27*). In contrast, the same mutation increased parasite susceptibility of 3D7 and NF54 lines to known antimalarials such as mefloquine (13.8-fold and 7.3-fold, respectively), dihydroartemisin (4.2-fold and 1.4-fold, respectively), and lumefantrine (5.4-fold in NF54; the 3D7 line was not tested). In addition, multicopy amplification of the *pfmdr1* region was observed in all 3D7 clones that had acquired resistance to MMV009063 (eight of eight clones; average amplification, 10-fold), MMV019017 (four of four clones),MMV665882 (five of five clones), and MMV665789 (seven of seven clones) (table S10). A nonsynonymous SNV was also discovered in *pfmdr2* (K840N in response to compound MMV665789). In addition, mutations were detected in the gene encoding a plasmamembrane– localized ABC transporter (*pfmrp2*;PF3D7_1229100) (*28*) in a clone that was resistant to imidazolopiperazines (selected with KAD707), as well as in two clones resistant to atovaquone. Changes in pfmrp2 transcript levels have been associated with levels of *P. falciparum* resistance to quinoline drugs (*29*), including chloroquine andmefloquine. A SNV was found in the ATP-binding cassette of *PfMRP2* at amino acid positionD976N in the KAD707-resistant clone, whereas an A403P change in the transmembrane domain and a frameshift mutation (N1974fs) were detected in the atovaquone-resistant lines. Subsequent testing identified clones with cross-resistance to mefloquine, consistent with *pfmdr1* amplification (fig. S5).

## Newly discovered drug-resistance genes

Knowing the identity of genes that impartmultidrug resistance is important for the design of new drugs, understanding how existing therapeutics can lose their efficacy in clinical settings, and identifying likely causative alleles in genomewide association studies. We observed that particular genes were mutated repeatedly in response to diverse compounds, indicating that they are more likely to be mediators of pleiotropic drug resistance. One such candidate is the putative ABC transporter encoded by *pfabcI3* (PF3D7_0319700) (*28*). Compounds that selected for mutations in *pfabcI3* (MMV020746,MMV023367,MMV024114, MMV029272, andMMV665939) are structurally diverse, and some are active across the parasite life cycle (*12*). The predicted gene product acquired point mutations [L690I, R2180G, R2180P (two times), and Y2079C] during resistance selections andwas central to 12 different amplification events on chromosome 3 (table S7 and Fig. 1C).

A previously unrecognized type of potential resistance mediator in this study was a predicted amino acid transporter of the SLC32 family of solute carrier proteins (encoded by *pfaat1*; PF3D7_0629500). Orthologs of this conserved protein are responsible for the transport of amino acids in synaptic vesicles in humans (*30*). Mutations in the encoding gene were associated with resistance to three diverse compounds: MMV007224 (-135I inframe insertion), MMV668399 (P380S, K238N, and V185L), and MMV011895 (F230L). This gene was found to be associated with levels of chloroquine resistance in *P. falciparum* in a genome-wide association study (*31*), and we found that the product of pfaat1 localizes to the *P. falciparum* digestive vacuole (fig. S6). Thus, the amino transporter encoded by *pfaat1* may play a role in the efflux of multiple drugs from the digestive vacuole, similar to PfCRT.

## Nonessential genes and drug activators

Because genes with premature stop codons (stopgained mutations) are unlikely to encode critical drug targets, genes with these mutations that were identified in our screen likely play a role in compound detoxification. The *P. falciparum* gene encoding the prodrug activation and resistance esterase (*pfpare*; PF3D7_0709700), which is annotated as a lysophospholipase, provides resistance to MMV011438 and pepstatin butyl ester (*32*). Another gene annotated as a lysophospholipase is PF3D7_0218600, which bears a FabD/lysophospholipase–like domain and harbors frameshift mutations in two independent clones (MALDA-Primaquine-PQG10 and MALDAPrimaquine- PQA11) that acquired blood-stage primaquine tolerance after 5months of exposure (fig. S2). This gene was notmutated in any of the other 261 clones and contained two of the eight codon-changing core mutations (three frameshift, fourmissense, and one stop-gained) in seven different primaquine-resistant clones.

In addition, the gene encoding the amino acid transporter (*pfaat2*; PF3D7_1208400) was found to have a stop codon in a clone (NMicro-GNF179- S2-3D7-2C) that is resistant toGNF179, an imidazopiperazine that is closely related to the clinical candidate KAF156 (*10*). *pfaat2* is predicted to contain 10 transmembrane domains and a pfam01490 amino acid transporter domain. The position- 903 stop mutation was found in concert with a splice acceptor intronic mutation in the gene encoding the acetyl coenzyme A transporter (*pfact*; PF3D7_1036800)—a gene whose disruption confers resistance to imidazolopiperazines (*15*). CRISPR-Cas9 introduction of the *pfaat2* L903 stop codon into GNF179-sensitive Dd2 parasites revealed that this mutation also confers resistance to GNF179 on its own (EC_50_ fold shift of 38 compared with the sensitive parent clone) (fig. S7).

## Transcription factors

Our study unveiled several resistance mediators that are likely to play a role in the parasite’s transcriptional response to drugs. Although there are no examples in _P. falciparum_, work in *S. cerevisiae* and other microbes has shown that mutations in transcription factors can often result in multidrug resistance (*33*). In our study, the most commonly mutated class of genes was that encoding the Apicomplexan AP2 transcription factor family (10 different variants observed in five different AP2 transcription factor–encoding genes, as well as several potential intergenic promoter mutations). In plants, AP2 transcription factors are involved in the cellular response to stress (*34*), and in *Plasmodium*, they regulate a variety of developmental transitions including commitment to sexual development and sporogony (*35*, *36*). The most prominent AP2 transcription factor in our set was encoded by PF3D7_0613800, which showed evidence of selection three times with three independent compounds (MMV665882, MMV665939, andMMV011438). In two cases, the same codon deletion (QMEGDNEMEGDNE197Q) was observed, and in one case, there was a nonsynonymous codon change (Q197E) at the same position. In addition, a region on chromosome 10 that encompasses a gene encoding another AP2 transcription factor [pfap2tf-10, also called *pfap2*-*i* (*37*); PF3D7_1007700] was amplified in nine clones (table S9). The amplification on chromosome 10 appeared in one primaquineresistant clone (consisting of only seven genes with an estimated four to five copies; *P* = 6.6 × 10^–5^), as well in clones resistant to GNF179, MMV008148, or MMV011438. Variants in the gene encoding the chromosome 6 transcription factor (PF3D7_0613800) were associated with resistance to quinine in genome-wide association studies (*38*).However, we still need to determine whether these changes are associated with multidrug resistance or are related to long-term in vitro culturing.

The Forkhead-associated domain is a phosphopeptide recognition domain found in some kinases and transcription factors (*39*). Mutations in a gene encoding an uncharacterized protein bearing this domain (*pffhd*; PF3D7_0909700) were found in all samples resistant to MMV668399 (*P* = 1 × 10^–18^ using an accumulative binomial distribution). Mutations included a M1V start-lost mutation, a K392 stop-gained mutation, L95R and G720E missense mutations, and a gene deletion (MALDAMMV668399- 2F2). Given that three of the mutations were predicted to result in loss of function, we hypothesize that this is a gene that inhibits drug action, although additional work needs to be done to characterize this further. These same resistant clones also contained mutations in the genes encoding the amino acid transporter (*pfaat1*; PF3D7_0629500) described above and the inorganic anion transporter (*pfsulp*; PF3D7_1471200) discussed below.

## New targets and compound target–inhibitor pairs

In addition to discovering genes involved in drug resistance, another goal of this study was to identify antimalarial drug targets to fill the need for alternative malaria treatments and advance the campaign to eliminate malarial infections in humans. In general, we considered enzymeencoding genes withmutations as potential drug target candidates, which was further supported if the mutated gene was compound-specific and docking and homologymodeling showedmutations in the catalytic site. A previously reported example is the set of BRD1095-resistant clones, which bear four different amino acid position changes (*40*) located in the predicted active site of the alpha unit of the cytosolic phenylalanyltRNA synthetase.

### Thymidylate synthase

**Fig. 3:**
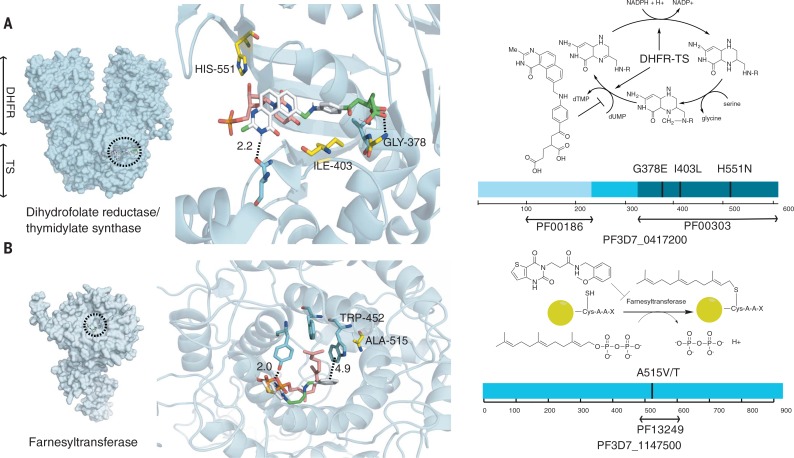
**A computational model of active antimalarial small molecules docked against their respective targets.** Schematics of the biochemical pathway, the compound, and conserved pfam domains are shown in the right panel. **(A)** MMV027634 occupies the thymidylate synthase active site. The benzoquinazoline head group of MMV027634 is stabilized by hydrogen-bonding within the dUMP active site of dihydrofolate reductase– thymidylate synthase (DHFR-TS). Further stabilization occurs at the tail with multiple hydrogen bonds to Gly378, mutation of which confers resistance to this compound. Mutations are all found in the thymidylate synthase portion of the molecule (http://pfam.xfam.org/family/pf00303). **(B)** MMV019066 and farnesyl pyrophosphate (FPP) are shown concurrently docked within the binding pocket of farnesyltransferase. A model of the P. falciparum farnesyltransferase beta subunit was constructed using the rat homolog (Protein Data Bank ID, 2ZIR) as a template. Despite substantial interspecies protein sequence variance, the FPP binding pocket is largely conserved (*69*). The preferential binding states of FPP andMMV019066 are shown competing for similar hydrophobic space. Resistance mutations are found in the squalene-hopene cyclase domain (http://pfam.xfam.org/family/pf13249). The yellow circle represents the rest of the donor protein to which the farnesyl group is attached. dTMP, deoxythymidine monophosphate; NADP+, nicotinamide adenine dinucleotide phosphate; NADPH, reduced form of NADP+; Me, methyl; R, benzoyl-L-glutamic acid. Single-letter abbreviations for the amino acid residues are as follows: A, Ala; C, Cys; D, Asp; E,Glu; F, Phe; G,Gly; H, His; I, Ile; K, Lys; L, Leu; M, Met; N, Asn; P, Pro; Q, Gln; R, Arg; S, Ser; T, Thr; V, Val; W, Trp; and Y, Tyr.

One likely target-inhibitor pair is dihydrofolate reductase–thymidylate synthase (encoded by *pfdhfr*-*ts*; PF3D7_0417200) and the benzoquinazolinoneMMV027634. Although dihydropyrimidine inhibitors of the bifunctional enzyme are wellknown antimalarials (e.g., pyrimethamine), all target the dihydrofolate reductase portion of the dual-function protein. We identified three different nonsynonymousmutations mapping to the thymidylate synthase portion of the bifunctional molecule in the MMV027634-resistant lines (Fig. 3A). Mapping of the mutations onto a published thymidylate *P. falciparum* crystal structure (*41*) showed that each mutation flanks the 2'-deoxyuridylic acid (dUMP)–binding site of the enzyme (Fig. 3A). Metabolomics (discussed below) and independent docking ofMMV027634 provided evidence that both substrates occupy the thymidylate synthase active site with a calculated affinity of –8.9 kcal/mol.

### Farnesyltransferase

Farnesyltransferase is encoded by another target gene (*pfftb*; PF3D7_1147500), which acquired two different mutations in amino acid 515 (A515V and A515T) in three clones resistant to the pyrimidinedione MMV019066 (Fig. 3B). Modeling shows that the mutation at amino acid 515 likely disrupts interactions between the thienopyrimidine and the farnesylation active site, resulting in a resistant phenotype. Previous work has shown mutations in the lipid substrate–binding site of farnesyltransferase in tetrahydroquinolineresistant parasites (*42*).

### Dipeptidyl aminopeptidase 1

An additional potential target gene encodes dipeptidyl aminopeptidase 1 (DPAP1) (*pfdpap1*; PF3D7_1116700), an exopeptidase that localizes to the digestive vacuole and cleaves amino terminal dipeptides from proteins or oligopeptides (*43*). Each parasite clone resistant toMMV029272, an arylurea, carried a mutation (L437S, L415P, or N62H) in *pfdpap1*. This gene is considered to be essential because it cannot be disrupted (*44*). All resistant clones also contained amplifications that encompassed the ABC transporter gene (*pfabcI3*; PF3D7_0319700), highlighting how resistance mechanisms can be found along with targets.

### Aminophospholipid-transporting P-type ATPase

As was previously reported for cladosporin and lysyl tRNA synthase (*13*), gene amplification events can occur around drug targets.We detected six CNVs in a genomic region on chromosome 12 that encompass a gene encoding a predicted aminophospholipid-transporting P-type ATPase (*pfatpase2*; PF3D7_1219600) (table S7 and fig. S3), previously named PfATP2. Tandem amplifications with approximate sizes of 8.7, 29, and 25 kbp were found in MMV007224-resistant clones, and amplifications of 102, 95, and 38 kbp were found in MMV665852-resistant lines. The 8.7-kbp amplification events encompassed only two genes—namely, the transporter gene and a truncated *var* pseudogene (PF3D7_1219500), indicating that the transporter is likely the target. This protein is closely related to PfATP4, an important antimalarial drug target. In the closely related rodent malaria parasite, *P. berghei*, this transporter was refractory to targeted gene deletion attempts, demonstrating its essentiality (*45*). MMV007224 and MMV665852 are structurally similar to one another and are both active against liver-stage parasites. *S. cerevisiae* orthologs of *pfatp2* encode the Dnf1/Dnf2 aminophospholipid translocases (flippases), which maintain membrane lipid asymmetry at the plasma membrane and contribute to endocytosis (*46*). In support of this activity, additional nonsynonymous variants in MMV007224-resistant lines were predicted to encode proteins involved in similar processes: Sec24, a component of the coat protein complex II (COPII) that promotes vesicle budding from the endoplasmic reticulum (ER), and Yip1, a protein required for fusion of these ER-derived vesicles with the Golgi. MMV665852 is a triclocarban, an antibacterial agent whose proposed mechanism is hypothesized to be inhibition of fatty acid synthesis, similar to triclosan, which was proposed to inhibit lipid and membrane function in *Plasmodium* parasites (*47*, *48*).

Last, in some cases, it can be difficult to determinewhether a gene encodes a promising target or is a resistance gene. For example, PF3D7_0107500 is annotated as encoding a lipid-sterol symporter in the resistance-nodulation-division (RND) transporter family. RND transporters are well characterized in Gram-negative bacteria, where they are involved in extruding toxins, exporting virulence determinants, and maintaining overall homeostasis (*49*). Although specific pumps in this family have been associated with multidrug resistance in bacteria (*50*), it is unclear what specific function the transporter encoded by PF3D7_0107500 has in *P. falciparum*. We detected five different SNVs in this gene in clones resistant to MMV028038,MMV019662, andMMV009108, in addition to 12 amplification events surrounding this gene in clones resistant to MMV028038 and MMV019662. Additional heterozygous SNVswere found in this gene in the MMV019662-resistant clones with chromosome 1 amplification events (table S14). MMV028038 and MMV019662 both have an amide bond,whereasMMV009108 does not. In addition, three different SNVs (D520Y, K615N, and I596M) were detected in an annotated inorganic anion transporter gene (*pfsulp*; PF3D7_1471200) in clones resistant to the pyrimidineMMV668399. The probability of randomly observing the same gene three times in six selections is extremely low (P = 4.0 × 10^–11^). PF3D7_1471200 encodes PfSulP, a protein of the SLC26membrane protein family (*51*)with11 transmembrane domains. In *P. falciparum*, this transporter has been localized to the surface of the parasite and is thought to play a role in the transport of anions across the parasite plasma membrane (*52*), although its role in drug resistance has not been well defined. Homology models with a fumurate transporter (*53*) showed that all mutations were located in the STAS domain, a cytoplasmic extension that in some species plays a role in signal transduction.

## Metabolomic profiling of compounds used to generate resistance

To functionally validate the modes of action associated with the targets predicted by resistance generation, metabolic perturbations associated with drug exposure (*54*) were assessed for 25 of the 37 compounds by using ultrahighperformance liquid chromatography–mass spectrometry (UHPLC-MS). Overall, the detected metabolic perturbations predict modes of action in accordance with the generated resistance mutations observed (figs. S8 and S9 and table S16). MMV008149, which resulted in a mutation in cytochrome b (*pfcytb*; mal_mito_3) (*12*), gives a classical response in the pyrimidine biosynthetic pathway with accumulation of *N*-carbamoyl- L-aspartate and dihydroorotate, indicative of disruption of cytochrome b or dihydroorotate dehydrogenase function. Similarly, as predicted by the thymidylate synthase resistancemutations and computational docking (Fig. 3),metabolomic analysis of MMV027634 revealed a clear signature in folate biosynthesis (figs. S8 and S9). MMV024114 has an analogous metabolic response and likely targets folate metabolism, although resistance mutations for this compound arose in *pfcrt* and *pfabcI3*, suggesting an adaptive effluxbased resistance by these parasites. Cluster analysis of the metabolic responses showed that compounds that yielded resistance mutations in the transporter genes *pfmdr1*, *pfaat1*, and *pflss* (MMV009108, ACT-451840, MMV665789, MMV019662, MMV007224, and MMV009063) had similar profiles. For many of the compounds that showed minimal metabolic perturbation (GNF179, MMV008149,MMV011438, and primaquine), resistance selection resulted in CNVs in *pfap2tf*-*10* [*pfap2*-*i* (*37*)]. Although not all compounds resulted in metabolic responses, the general concordance between metabolic changes and resistancemutations supports the combined use of these approaches for compound target identification.

## Discussion and conclusions

Our study represents a controlled examination of antimalarial drug resistance acquisition by *P. falciparum*. Prior studies examining the parasite’s genetic response during drug resistance development have evaluated the response only to known antimalarials (*31*, *38*, *55*, *56*) or have focused on coding mutations in one target gene in response to a single compound class (*8*, *13*, *32*, *40*, *57*–*60*). It is likely that the genes that are identified here will prove important in both clinical studies and the process of drug development.

One noteworthy finding from this data set is the high enrichment ofmutations under positive selection. We focused here on genes for which there were multiple lines of supporting evidence across our study; however, it is likely that other important genes were identified. Excluding genes contained within CNVs, the 57 singleton genes with nonsynonymousmutations encode potential druggable targets, such as guanosine triphosphatases, mRNA decapping enzymes, components of V-type ATPase complexes, kinases, and ubiquitin ligases (table S5). Although some of these nonsynonymous changes could have been maintained within the population by chance, some are plausible targets of selection. For example, although resistance to ACT-451840 is conferred by mutations in *pfmdr1* (*61*), a mutation was observed in the gene encoding a putative replication factor C subunit 4 (PF3D7_1241700) that results in a S42T amino acid change near the ATP-binding pocket in two independent ACT-451840–resistant clones (*61*). Replication factor C subunit 4 is the subunit ATPase in the clamp-loading DNA polymerase complex, is likely essential, and is a good drug target. Modeling shows that ACT-451840 could bind between subunits 1 and 4 of replication factor C, potentially disrupting ATPg ingress (fig. S10).Our findings also highlight the large number of uncharacterized genes in the *P. falciparum* genome and the need for further functional annotation.

Not all mutations discovered in this study will confer resistance—they may compensate, specifically or nonspecifically, for fitness losses that result from a resistance-conferring mutation. Alternatively, they may be associated with adaptation to long-term in vitro culture. Genome editing or creating recombinant lines may be used to confirm that alleles provide resistance (*8*, *15*, *27*, *40*, *58*, *62*, *63*). On the other hand, given the multigenic nature of resistance, it may be difficult to recreate a resistance phenotype (*7*). For example, the nonsynonymous changes that emerged in phenylalanine tRNA ligase during development of resistance to BRD1095 were accompanied by copy number changes at unrelated sites, which potentially explains why attempts at validation through genome editing were unsuccessful.

Beyond changes in coding sequences, the low frequency at which noncoding variants were found in our data set suggests a possible role.We identified only 21 silent mutations within genes that are plausible drug targets, including those encoding protein kinase 4,Ark3 kinase, cytochrome c oxidase 3, a histone-lysine N-methyltransferase, a putative serine threonine protein kinase, a DNA helicase, and an 18S ribosomal RNA. Several of the genes with silent mutations were highly expressed, including the cytochrome c oxidase subunit 3 gene, in which the mutation substitutes a rare codon for a frequently used one [acA (23.8% usage for threonine) to acG (3.6%)]. Silent mutations in human *mdr1* can confer resistance to cancer drugs owing to altered protein folding (*64*). Further work will be needed to establish whether these silent substitutions play a role in drug resistance in P. falciparum. Similarly, intergenic and intronic mutations were also common. Although in most cases, resistance was explained by the presence of nonsynonymous coding mutations in the target or resistance genes, intergenic mutations were also common. Likewise, intron variants were more likely to be found in the core genome than in subtelomeric regions (64:5), suggesting a possible functional role.

Last, it is likely that among the genes that we identified, several may contribute to clinical resistance at some level. Although pharmacokinetics are different in vivo than in vitro, we repeatedly rediscovered mutations in genes important for clinical resistance. It is therefore likely that previously unidentified genes are candidates to be under selection in clinical isolates. Mutations in 3247 clinical *P. falciparum* isolates reveal that four mediators of resistance (the ABC transporter gene *pfabcI3*, the putative amino acid transporter gene *pfaat2*, the AP2 transcription factor gene *pfap2tf-6b*, and the farnesyltransferase gene *pfftb*) have nonsynonymous-to-synonymous ratios greater than 2:1, suggesting that they are under positive selection (*65*) (table S15). However, the role of these genes in clinical drug resistance is unknown. In addition, our data highlight the importance of CNVs in conferring multidrug resistance and suggest that high-coverage genome sequencing of clinical isolates will provide information on selective pressures in the field (*66*). It is notable that we were able to identify a likely target or resistance gene for every compound for which resistant parasites were generated. The haploid genome and the lack of transcriptional feedback loops suggest that *P. falciparum* is a particularly good model for both target identification and resistome studies. Our characterization of the chemogenetic landscape of *P. falciparum* will guide the design of small-molecule inhibitors against this deadly eukaryotic pathogen.

## Supplementary Material

Mapping the malaria parasite druggable genome by using in vitro evolution and chemogenomics

Mapping the malaria parasite druggable genome by using in vitro evolution and chemogenomics

## SUPPLEMENTARY MATERIALS

www.sciencemag.org/content/359/6372/191/suppl/DC1

Tables S1 to S16

References (70–93)
