# Supplementary material for: Mapping the malaria parasite druggable genome by using in vitro evolution and chemogenomics
Source: Science. 2018 Jan 12;359(6372):191–9. doi: 10.1126/science.aan4472 (PMC5925756; doi:10.1126/science.aan4472)
Supplement: Mapping the malaria parasite druggable genome by using in vitro evolution and chemogenomics [file Science-359-191-s001.pdf]

www.sciencemag.org/content/359/6372/191/suppl/DC1

Supplementary Materials for

Mapping the malaria parasite druggable genome by using in vitro evolution and chemogenomics

Annie N. Cowell, Eva S. Istvan, Amanda K. Lukens, Maria G. Gomez-Lorenzo, Manu Vanaerschot, Tomoyo Sakata-Kato, Erika L. Flannery, Pamela Magistrado, Edward Owen, Matthew Abraham, Gregory LaMonte, Heather J. Painter, Roy M. Williams, Virginia Franco, Maria Linares, Ignacio Arriaga, Selina Bopp, Victoria C. Corey, Nina F. Gnädig, Olivia Coburn-Flynn, Christin Reimer, Purva Gupta, James M. Murithi, Pedro A. Moura, Olivia Fuchs, Erika Sasaki, Sang W. Kim, Christine H. Teng, Lawrence T. Wang, Aslı Akidil, Sophie Adjalley, Paul A. Willis, Dionicio Siegel, Olga Tanaseichuk, Yang Zhong, Yingyao Zhou, Manuel Llinás, Sabine Ottilie, Francisco-Javier Gamo, Marcus C. S. Lee, Daniel E. Goldberg, David A. Fidock, Dyann F. Wirth, Elizabeth A. Winzeler*

*Corresponding author. Email: ewinzeler@ucsd.edu

Published 12 January 2018, *Science* **359**, 191 (2018)
DOI: 10.1126/science.aan4472

This PDF file includes:

Materials and Methods
Figs. S1 to S10
Tables S7 to S9, S14, and S15
Captions for Tables S1 to S6, S10 to S13, and S16
References

Other Supplementary Material for this manuscript includes the following:
(available at www.sciencemag.org/content/359/6372/191/suppl/DC1)

Tables S1 to S6, S10 to S13, and S16

Table of Contents

Author Contributions 3

Materials and Methods.. 4

Figure S1 13

Figure S2 14

Figure S3 15

Figure S4 16

Figure S5 17

Figure S6 18

Figure S7 19

Figure S8 20

Figure S9 21

Figure S10 22

Table S7 23

Table S8 24

Table S9 25

Table S14 26

Table S15 27

Additional Data (Captions for Tables S1-S6, S10-S13, S16)... 28

Additional References (70-73)……………..……………………………………………….30

Author Contributions

E.A. Winzeler and A.N. Cowell analyzed data, compiled tables and wrote the manuscript. Selections were conducted by G.M. Lamonte, M. Abraham, C. Reimer, V.C. Corey, E.L. Flannery, E. Sasaki, O. Fuchs, A.K. Lukens, E. Istvan, O. Coburn-Flynn, M.C.S. Lee, T. Sakata-Kato, P. Magistrado, C. Teng, S. Bopp, P. Gupta, M. Linares, M.G. Gomez-Lorenzo, I. Arriaga, M. Vanaerchot, N.F. Gnadig, J.M. Murithi, and V. Franco. Killing rate assays were performed and analyzed by G.M. Lamonte, M. Abraham, C. Reimer, E.L. Flannery, S.W. Kim, C.H. Teng, V.C. Corey, E. Sasaki, P. Gupta, E. Istvan, A.K. Lukens, T. Sakata-Kato, P. Magistrado, S. Bopp, M. Linares, I. Arriaga, M.C.S. Lee, V. Franco, and M.G. Gomez-Lorenzo. Circos plot and chemoinformatics analysis was done by O. Tanaseichuk, Y. Zhong, and Y. Zhou. Docking and homology modeling was done by M. Abraham. Sequencing libraries were prepared by S.W. Kim and V. Corey. WGS analysis was performed by A.N. Cowell, V. Corey, and E. L. Flannery. R.M. Williams developed the copy number variant calling pipeline. P. Willis provided compounds. D. Siegel provided information regarding compounds. S. Ottilie coordinated the selection process and exchange of data. Pedro Moura contributed Figure S6. L.T. Wang analyzed CNVs by PCR assay. A. Akidil and S. Adjalley performed and analyzed the CNV PCR data. M. Llinás, E. Owen, H.J. Painter performed the metabolomics assay and analyzed the data. F.J. Gamo, M.C.S. Lee, D.E. Goldberg, D.A. Fidock, D.F. Wirth, and E.A. Winzeler supervised the project, provided funding and resources, and helped edit the manuscript. All authors approved the final manuscript.

Materials and Methods

Compound origin and computational clustering

Compounds were all publically available and obtained from a variety of sources, including the MMV malaria box[12], the GlaxoSmithKline Tres Cantos Antimalarial Set (TCAMS)[5], the University of Dundee, the Broad Institute's Diversity Oriented Synthesis libraries, and the GNF malaria box[7]. A putative target matrix for all compounds were assembled by calculating the total occurrence of all mutations (CNV, SNV and indel) for a given compound-target pair as matrix elements. The matrix was hierarchically clustered on both targets dimension and compound dimensions. Similarity between compound pairs was examined using target and or resistance profile similarities and structural similarity based on ChemAxon topological fingerprints (ChemAxon, Kft.).

Generation of compound-resistant clones

Parasites were cultured in RPMI1640 media supplemented with 0.5% Albumax II, 4.3% human serum, 25 mM Hepes, 25 mM $NaHCO_3$, 0.36 mM hypoxanthine and 100 ug/mL gentamicin and incubated at 37°C in an atmosphere of 3% O_2, 4% CO_2 and 93% N_2. Cultures were maintained in leukocyte-depleted red blood cells at 2.5% hematocrit. Compound-resistant parasites were developed using either a high-pressure intermittent, a step-wise, or a constant method of compound exposure as previously described[12]. Briefly, for the high-pressure intermittent method, approximately 1-2x 10^9 clonal aliquots of *P. falciparum* Dd2 or 3D7 strains were treated at a concentration of 3-10x EC_{50} until parasites were no longer visible by microscopy. After parasites grew back to a parasitemia of approximately 2%, compound pressure was reinitiated. The constant method of compound exposure is similar to this method, except that the compound pressure is never removed from the parasite culture. For the step-wise method, 1-2x 10^8 parasites were cultured in triplicate and exposed to increasing concentrations of compound, resulting in a reduced growth rate of approximately 50%. Once parasites achieved parasitemias of 6-8%, they were diluted to 1% parasitemia and compound concentration was increased again, generally by 10%. Cultures were maintained until clones demonstrated a reproducible

EC_{50} fold shift of >3X. At the end of successful selection, parasites were cloned in 96-well plates by limiting dilution[70].

To assess the half maximal (50%) effective concentration (EC_{50}) of the compound being evaluated, artemisinin and chloroquine were determined in dose-response format using a SYBR Green-I based cell proliferation assay as previously described[7]. Briefly, parasites were incubated in 96-well format with exposure to a 12-point dilution series of the compound of interest. Following incubation for 72 hours, parasites were lysed; DNA was stained using SYBR Green fluorescence and I was measured at 535 nm on an Envision plate reader (Perkin Elmer, Waltham, MA) after excitation at 485 nm. EC50 assays were repeated three times and averaged to get the final EC50 value. The EC50 values for each clone were compared to that of the corresponding sensitive parent clone to determine fold-shift changes. Four parameter dose-response curves were fitted and log(EC_{50}) values calculated using Prism (Graphpad Prism, La Jolla, CA).

For each compound, at least three independent, stable, resistant clones were available for sequence analysis. 173 compound-resistant clones or isogenic parents were derived from the 3D7 *P. falciparum* strain background, 84 were from a Dd2[71] strain background, and 5 were from a 7G8 strain background[27, 72, 73]. The 3D7 line is mostly drug-sensitive, although it does convey resistance to sulfadoxine and bears an amplification of GTP cyclohydrolase[74], a gene in the folate biosynthesis pathway. The Dd2 line is multi-drug resistant, originating from an Indochina III/CDC isolate, and containing mutant *pfcrt* sequence including the chloroquine resistance marker K76T, as well as amplifications in *pfmdr1* and GTP cyclohydrolase that decrease parasite susceptibility to mefloquine, lumefantrine and pyrimethamine[71]. The 7G8 strain is resistant to chloroquine, amodiaquine, and pyrimethamine[72, 73, 75].

Mefloquine cross-resistance testing

Dose-response experiments were performed in triplicate with synchronous, young ring-stage cultures (1-1.2% starting parasitemia) as previously described[32]. Parasitemias (percentage of total erythrocytes infected with parasites) were measured approximately 70-80 hours post compound addition by nucleic acid staining of iRBCs with 0.8 mg/ml acridine orange in PBS. Growth was normalized to parasite cultures with carrier only

(DMSO). Chloroquine was used as a no growth (kill) control. Inhibition data were fit to a sigmoidal dose-response curve using Graphpad Prism 5.0.

Library preparation and analysis of sequenced samples

Genomic DNA (gDNA) was obtained from the sensitive parent clone and compound-resistant clones by washing infected RBCs with 0.05% saponin and isolating the gDNA using a DNeasy Blood and Tissue Kit (Qiagen), following the standard protocols. Sequencing libraries were prepared with the Nextera XT kit (Cat. No FC-131-1024, Illumina) using the standard dual index protocol and sequenced on the Illumina HiSeq 2500 with a RapidRun mode, sequencing 100 base pairs deep on either end of the fragments. Altogether, 204 clones were resequenced to an average of 80.3x coverage using 100 base pair paired-end reads. 58 previously published sequences were downloaded from the National Center for Biotechnology Information's (NCBI) sequence read archive and analyzed in parallel.

Following sequencing, reads were aligned to the *P. falciparum* 3D7 reference genome (PlasmoDB v. 13.0), following the Platypus pipeline as previously described[76] (for SNVs and CNVs), with the exception that single nucleotide variants (SNVs) and insertion/deletions (INDELs) were called with the Genome Analysis Toolkit's (GATK) HaplotypeCaller[77-79]. To identify valid variants, mutations were filtered using general recommendations from GATK. SNVs were filtered out if they met the following criteria: ReadPosRankSum >8.0 or <-8.0, QUAL<500, Quality by Depth (QD)<2.0, Mapping Quality Rank Sum <-12.5, and filtered depth (DP) <7. Indels were filtered out if they met the following criteria: ReadPosRankSum <-20, QUAL<500, QD<2, and DP<7. Following the initial filtration, we removed mutations where read coverage was <5 and/or where mixed read ratios were >0.2 (reference/total reads) across all samples. Variants were annotated using SnpEff[67]. Identified variants were further filtered by comparing those from resistant clones to the parent clone, such that a K76T mutation in *pfcrt* would not be called if this mutation were in the parent Dd2 clone. Since all parasites lines were cloned before sequencing, only homozygous variant calls were retained.

CNV detection

Given that *P. falciparum* is 90-95% AT-rich in intergenic regions[23], only genic coverage was analyzed, as highly AT-rich regions have a larger variability in coverage due to PCR-amplification bias. Alignment confidence is also reduced in intergenic regions due to AT-repeat segments, and therefore analyzing only genic regions provides the most robust CNV analysis. The data were analyzed in three groups based on predicted genetic background (e.g. Dd2-based (83 samples), 3D7-based (173 samples) or 7G8 (5 samples)), which allowed the identification of CNVs around *pfmdr1* in 3D7 parasites. Average coverage was calculated with GATK's DiagnoseTargets tool, supplying gene lengths and locations as the analyzed intervals. *var, rifin,* and *stevor* family genes were removed from the interval list, as these genes are known to have a high variability in copy numbers and cause misalignment issues[80-82].

The R programming language[83] was used to construct a data analysis workflow for the determination of copy number variants based on differential DNA sequence coverage across the *P. falciparum* genome. The pipeline R functions are available as an RStudio project from https://bitbucket.org/rwillia2001/plasmodium_cnv_analysis and are distributed as open source under the terms of the GNU General Public License. Read coverage was normalized for each set by (1) log transforming the data and centering genes to the mean (normalize genes) and (2) center sequencing samples to the sample means (normalize samples). Amplified genes were identified if they showed a 2-fold increase above the mean coverage. We deemed a 4-gene unit as the minimal length for a CNV of biological interest since CNVs of interest typically cover genomic windows of at least 2 genes, and have recombination breakpoints in the neighboring regions. To distinguish between normal coverage fluctuation and regions more likely to harbor biologically significant alterations, we filtered for regions with a higher proportion of adjacent genes with a greater than 2-fold increase. As CNV length increases they become highly unlikely to be observed by random chance, as shown by permutation of the data and re-running the CNV filtering function (see permutation R "sample" function). Since these low stringency filters captured small mitotic recombination events that arise during long-term growth[14], we further required CNVs to be in the core genome and to have greater than 3x coverage relative to the average over the length of the CNV.

Each CNV region in a sample was given a t-test p-value significance by comparison between the CNV region read depth vector and the same region vector in the other samples. Levene's test was used to investigate "variances equal" and probability values were calculated using a Benjamini-Hochberg corrected t-test (one-tailed, variances equal) of the average normalized coverage for all genes in the interval for the *P. falciparum* clone relative to the average normalized coverage for all genes in the interval for all other clones. To establish the false discovery rate, read coverage was randomized as a function of chromosome position and probability values were calculated. Applying similar filters (average read coverage > 3x, $p<0.001$), we identified only 8 CNVs in the permuted 3D7 set and 1 in the Dd2 permuted set. Six CNVs that failed the quality threshold but were visually identifiable and present in other clones in the selection group were also added for a final set of 159 (Figure 1, Table S8). CNV deletion events were determined by looking for a significantly lowered normalized sequence coverage of < (-0.05), and following the same workflow as described above.

CNV detection by quantitative real-time PCR and junction PCR assay

We designed primer pairs to amplify the region that crosses CNV junctions using the sequences of reads with higher than expected insert sizes that flank regions with significantly increased coverage, which were identified manually using the Integrated Genome Viewer (IGV)[25]. Since the CNV junctions fall across the end and beginning of the amplified region, one would not expect to find this sequence in the parasite genomes that do not harbor that CNV. The primer pair 5'- TGTAAATTTTCATTTTTCCCA-3' and 5'- ATGTGCGTATTTAATAGATT-3' was designed to amplify the junctions of the CNV surrounding *pfabcI3* in the MMV029272-resistant clones. The primer pair 5'-TGTAAGTATGAATGTCATGTT-3' and 5'- ACTTCAAATATGCGTAAAAGC-3' was designed to amplify the junctions of the CNV surrounding *pfmdr1* in MMV665789 clones. A primer pair designed to amplify a 104 base pair amplicon of the *P. falciparum* 18s ribosomal RNA (PF3D7_0112300) gene (5'-AACCTGGTTGATCTTGCCAGT-3' and 5'-AATGAGCCGTTCGCAGTTTC-3') was used as a positive control. The PCR reaction was carried out using PrimeSTAR Max DNA polymerase (Takara) in a total volume of 20 µl in a thermocycler with the following settings: 95°C for 10 minutes, followed by 40

cycles of 95°C for 15 seconds, 57°C for 3 seconds, and 60°C for 60 seconds, then a hold at 4°C. PCR products were run on a 1.5% agarose gel.

For copy number quantification by real-time PCR, assays were performed in a LightCycler 480 instrument (Roche Diagnostics, Penzberg, Germany) with PowerUp SYBR Green master mix (2X) (ThermoFisher). Triplicate samples were run in parallel in 96-well plates in a total reaction volume of 20 µl comprising 0.04 ng/µl of genomic DNA and 0.3 µM of each primer. Target gene copy number was assessed relative to the control gene, ß-tubulin (PF3D7_1008700), using a standard curve generated from serial 10-fold dilutions of 3D7 genomic DNA. Primer sets were designed and used for the amplification of the *pfatp2* (PF3D7_1219600) gene (5'-TCGTTTGTGCTCCTAGTTACTG-3'and 5'-AACGAAGAGACTGAACGAGATATAC-3'), the *pfabci3* (PF3D7_0319700) gene (5'-CAGGTGAATAGTCAAATGTGTTTAAGG-3' and 5'-GTCGAAGAGGTATCATGGGAATG-3'), and the *pfpi4k* (PF3D7_0509800) gene (forward, 5'-TGCTGAGCCAGATATTGATCTAC-3' and 5'-CACAACAAGCCACTATACATCTTC-3'). Primers for β-tubulin amplification are as follows (5'-CGTGCTGGCCCCTTTG-3' and 5'-TCCTGCACCTGTTTGACCAA-3'). The qPCR conditions were: 5 min at 95°C, followed by 45 cycles of 10s at 95°C, 15s at 56°C, and 10s at 72°C.

Computational enrichment analysis

Gene lists were loaded onto PlasmoDB (release 27) to search for gene enrichment[84]. Gene enrichment analyses were generated for each individual ontology group (biological process, molecular function, and cellular component), utilizing both the InterPro domain database and the Annotation Center (which downloads GO annotations from sequencing centers, including GeneDB and JCVI) as GO association sources. Additionally, a p-value cutoff of 0.05 was required to select enriched annotations.

PfAAT localization

Parasites expressing an AAT1-mRFP fusion protein were generated by transfecting $Dd2^{attB}$ parasites[85] with pINT and pM plasmids. The pINT plasmid has been described elsewhere[86]. The pM plasmid was generated by replacing the *pfcrt* cassette of the

previously generated pM-BSD-hrp3-crt(GC03)-mRFP plasmid[87] with PCR-amplified *pfaat1* using the AvrII and BglII sites. The *attP* site was introduced by digestion from the pLN-ENR-GFP plasmid[85]. Ring-stage Dd2^{attB} parasites were transfected with 50 µg of each plasmid in a 0.2 cm cuvette using the Gene Pulser II electroporator (Bio-Rad) at 0.31 kV, 950 µF, and maximum resistance. Transfected parasites were selected with 125 µg/ml G418 and 2.5 nM blasticidin until recombinant parasites emerged. Live intra-erythrocytic Dd2 parasites expressing the PfAAT1-mRFP fusion protein were fixed on MatTeck glass bottom petri dishes using a Fibrinogen/Thrombin clot, as described elsewhere[88]. Parasites were then stained with Hoechst 33342 and subsequently imaged at a 90x magnification using an Olympus IX81 inverted microscope with a 12 bit Cooke Sensicam QE cooled CCD camera.

Homology models and protein structure

The crystal structure of *P. falciparum* dihydrofolate reductase-thymidylate synthase (4DPD; 2.50Å) was used for docking[89]. For the remaining proteins, homology models were constructed using SWISS-MODEL using the following templates; replication factor C (1SXJ; 2.85Å); farnesyltransferase (PF3D7_1147500), rat Fase template (2ZIR; 2.40Å), and phenylalanine--tRNA ligase (PF3D7_0109800); human phe-tRNA synthetase template (3L4G; 3.30Å).

CRISPR/Cas9 Genome Editing

The PF3D7_1208400 (*pfaat2*) mutation identified via *in vitro* selection was confirmed by introducing those mutations into the parental Dd2 line using a CRISPR/Cas9 system[27]. Cas9 was expressed from a pDC2-based human *dhfr* plasmid, along with a sequence encoding the guide RNA (gRNA). gRNA expression was driven by the T7 promoter via co-expression of the T7 RNA polymerase from a pDC2-bsd plasmid. A donor template with homology to the target site was also supplied on the same pDC2-bsd plasmid containing T7 RNA polymerase, and contained both the desired nucleotide replacement and also silent mutations in the gRNA site to prevent cleavage of the donor or the modified genome. These plasmids were electroporated (310kV, 950 µF) into sorbitol-synchronized ring-stage Dd2 parasites. Parasites were first selected for 6 days with 1.5 nM WR99210 and 2 µg/ml blasticidin, and then with 5x EC_{50} GNF179. The time to recover polyclonal

parasite lines was approximately 28 days, after which transformed parasite lines were cloned by limiting dilution. Successful editing was validated by PCR and Sanger sequencing, and validated parasite lines with the desired mutation were assayed for resistance to GNF179 using the SYBR Green I assay described above.

The guide RNA sequence (listed as gRNA+PAM) used for PF3D7_1208400 was TCAGGCAAAAAAGTATAGG, while donor templates consisted of a ~1 kb region around the mutation of interest (F primer: AACAGTCATGGGATTTGAAAAA, R primer: TGCCCATGTTAAATTTTGTTT). Mutations in the donor template, both for the gRNA target site and the desired SNV, were introduced via the Quikchange II kit (Agilent Technologies).

Docking studies

All docking simulations were performed using AutodockVina (v1.1.2)[90]. Autodock Tools (v1.5.6) was used to prepare the ligands and grid space of each protein[91]. Visual analysis of energetically favorable binding states was performed using PyMOL (v1.8.4.0)[92].

Metabolomic profiling

To determine the metabolic response of trophozoite stage *P. falciparum* to select compounds, we performed whole cell metabolite extractions that were measured and quantified using metabolomic analysis by ultra-high precision liquid chromatography mass-spectrometry (UHPLC-MS). This method allows for the direct observation of *in vivo* effects on parasite metabolism and aids in the determination of potential modes of action (MoA). Magnetic separation of mature trophozoites (24-36 hpi) was carried out as previously described [54] using an in-house constructed magnetic cell fractionation system. Upon purification, the number of parasitized red blood cells were quantified using a hemocytometer and parasites were transferred to 6-well plates at a concentration of $1x10^8$ cells/well. Parasites were allowed to recover under standard culture conditions at 0.5% hematocrit for 1-2 h before experimentation. Following recovery, parasites were either untreated, incubated with each compound (10x IC_{50}), or incubated with atovaquone (10xIC_{50}), in triplicate at 37^oC with appropriate gas mixture for 2.5 hours. After

incubation, cells were then collected by centrifugation and hydrophilic extraction of intracellular metabolites was performed using 90% methanol containing isotopically-labeled aspartate (0.5 μM) as an internal standard. Metabolite extracts were then measured by UHPLC-MS as previously described and data were analyzed using MetaPrints as previously described [54]. The metabolite data (Table S16) for each drug treatment was directly compared to a mock-untreated control and the resulting fold-changes were hierarchically clustered based on a Pearson distance (Fig. S8) and visualized using MetaPrint self-organizing maps [54].

Figure S1

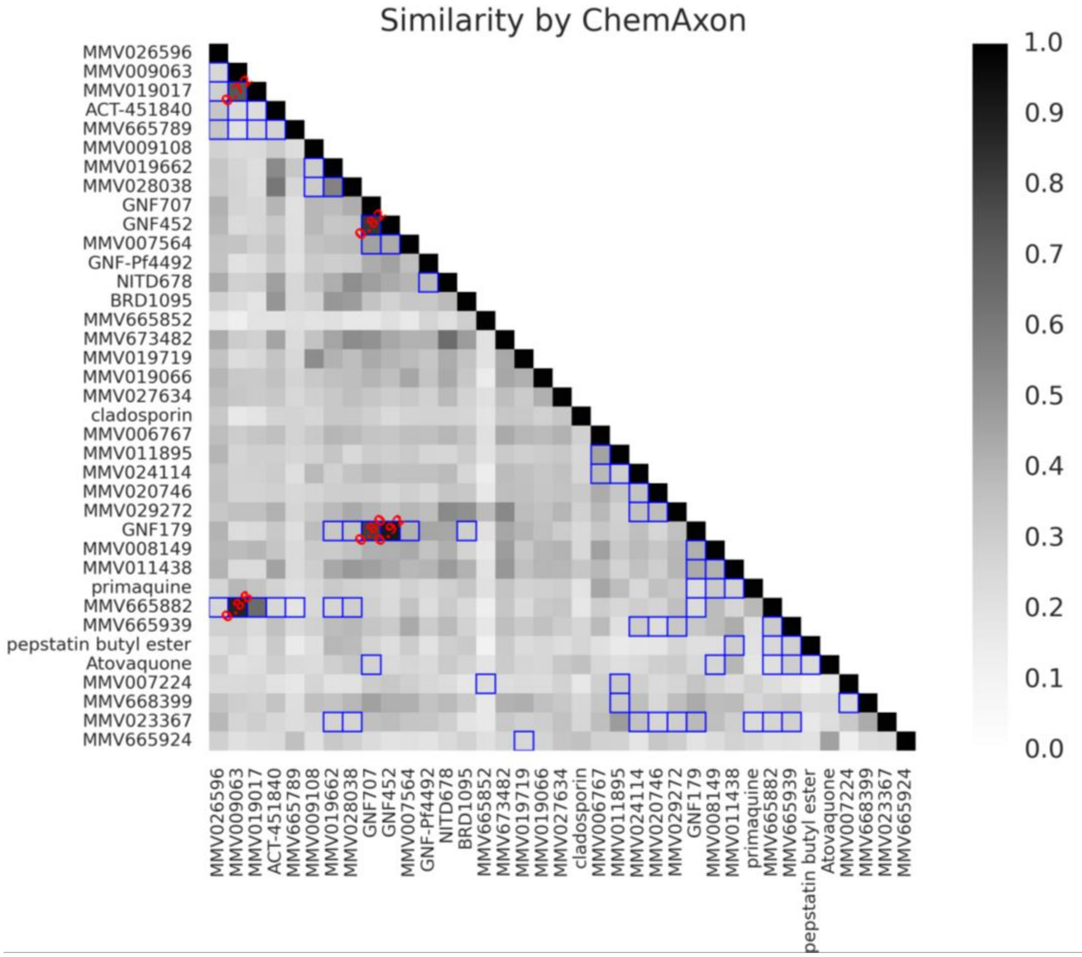

Fig. S1. Compound structure similarity heatmap constructed using ChemAxon fingerprints. The order of compounds is adopted from Figure 2. The red text highlights the compound pairs that have Tanimoto similarity coefficients of greater than 0.7. The blue boxes highlight compound pairs that share at least one common mutation (Pearson's correlation coefficient >= 0.6). For example, amplification of the *pfmdr1* region was observed in all 3D7 clones that acquired resistance to three closely related carbazoles: MMV009063, MMV019017, and MMV665882. In addition, independent clones resistant to imidazolopiperazines (GNF452, GNF707, GNF179) and the closely related compound, MMV07564, acquired mutations resulting in coding changes in the cyclic amine resistance locus (*pfcarl*;PF3D7_0321900).

Figure S2

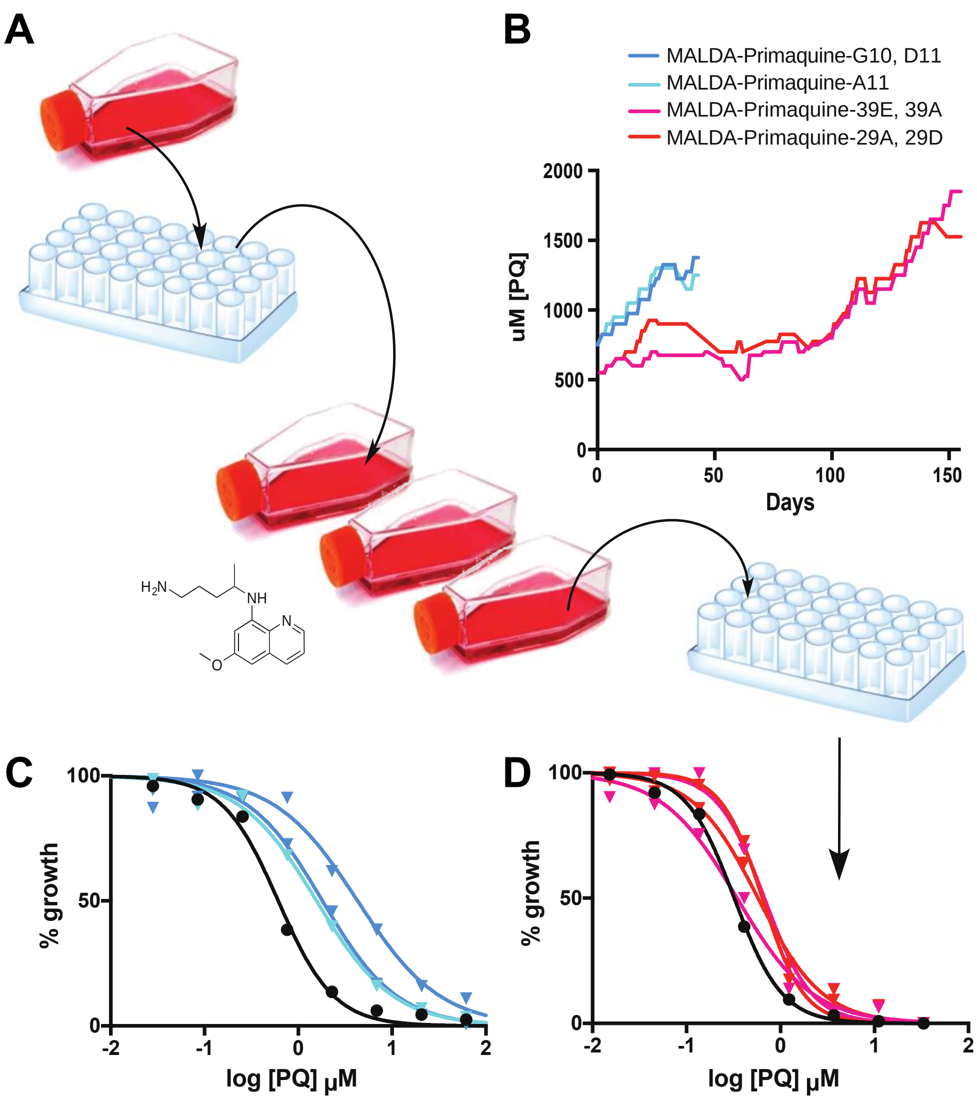

Fig. S2. Generation of primaquine resistance in *P. falciparum* using a step-wise method of compound exposure. (**A**) *P. falciparum* (Dd2 strain) clones were generated using limiting dilution. Three independent clones were cultured in separate flasks in the presence of increasing concentrations of primaquine and cloned again prior to whole genome sequencing analysis. (**B**) The flasks of *P. falciparum* were exposed to increasing concentrations of primaquine for 45 or 150 days. EC_{50} curves for the *P. falciparum* primaquine resistant clones in the 45-day exposure group (MALDA-Primaquine-G10, D11, and A11) (**C**) and the 150 day exposure group (MALDA-Primaquine-39E, 39A, 29A, and 29D) (**D**) demonstrate an increase in the EC_{50} value in the exposed clones (triangles, colored lines) compared to the sensitive parent *P. falciparum* clone (circles, black lines).

Figure S3

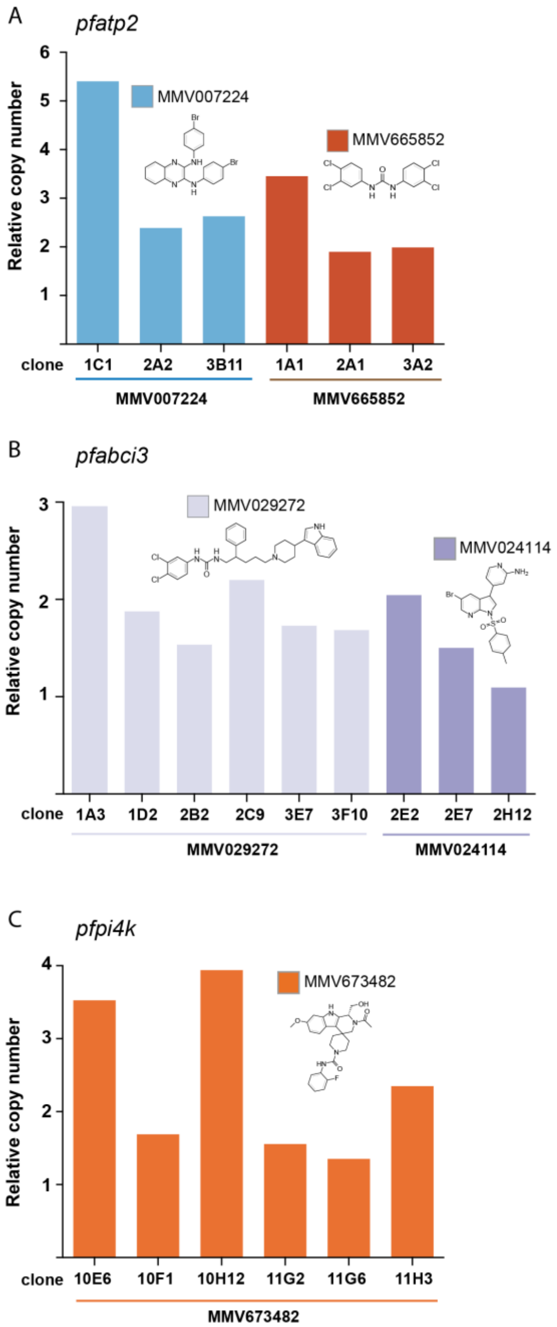

Fig. S3. Detection of copy number variants (CNVs) using real-time quantitative PCR. We tested 21 *P. falciparum* clones for CNVs encompassing 3 genes of interest detected in whole-genome sequencing data: (**A**) the aminophospholipid-transporting P-ATPase (*pfatp2*; PF3D7_1219600) (**B**) ABC transporter I family member 1 (*pfabcI3*; PF3D7_0319700), and (**C**) phosphatidylinositol 4-kinase (*pfpi4k*; PF3D7_0509800). Increased copy number of these genes relative to the β-tubulin gene was detected in the expected clones.

Figure S4

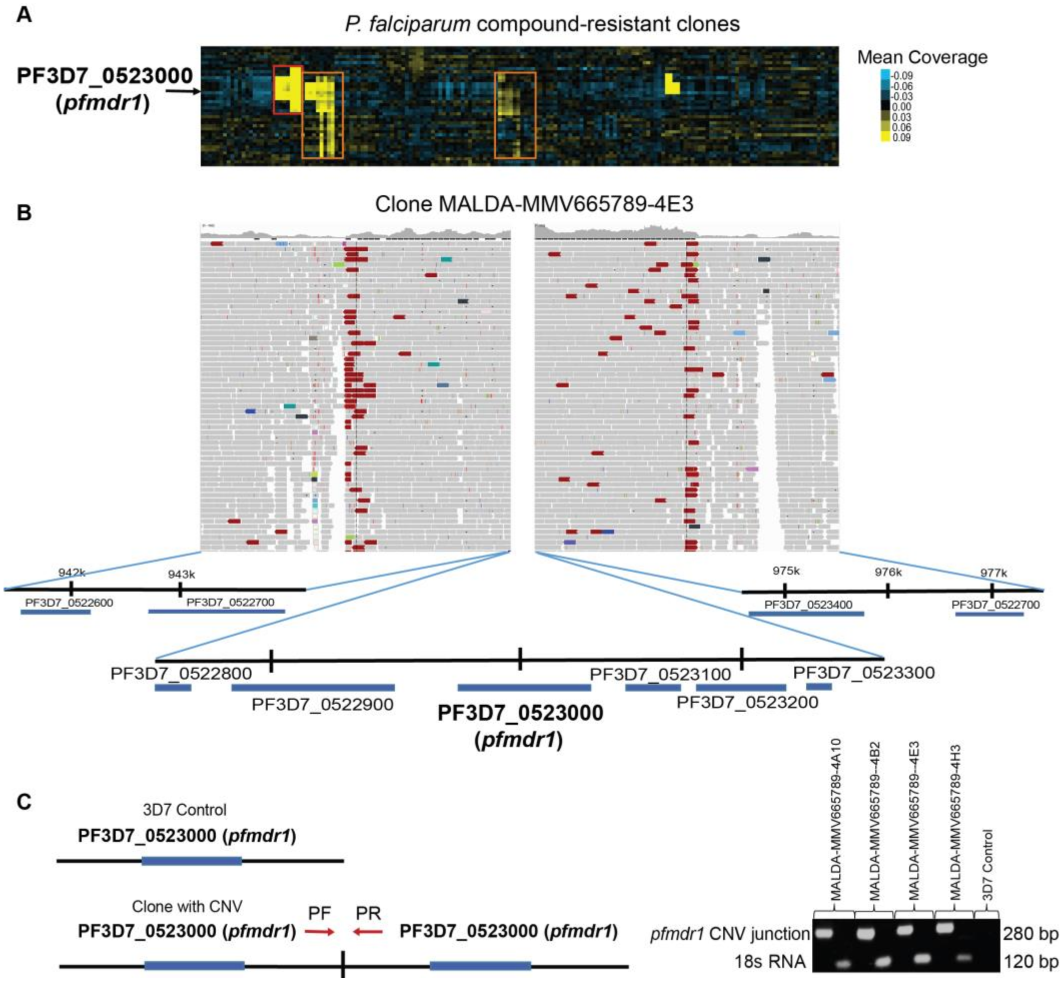

Fig. S4. Confirmation of copy number variants (CNVs) in *P. falciparum* compound-resistant clones. (**A**) Heat map of normalized gene coverage across all 262 sequences (visualized with Java Treeview[93] shows a subset of 41 genes (PF3D7_0521700-PF3D7_0525600) surrounding *pfmdr1* (PF3D7_0523000), with each column representing one clone and each row representing one gene. Genes with higher coverage are brighter yellow according to the color bar which shows color for mean coverage. The amplification surrounding *pfmdr1* in clones resistant to compound MMV665789 is outlined in red and the two groups of clones resistant to compounds classified as carbazoles (MMV009063, MMV665882) are outlined in orange. (**B**) Junctions of this CNV in clone MALDA-MMV665789-4E3 are visualized using the Integrated Genome Viewer (IGV)[25]. Outward facing reads with insert sizes that are larger than expected (~33,000 bp) on either end of the region of amplification are highlighted in dark red. (**C**) The CNV junction PCR assay uses primers (PF – forward primer; PR – reverse primer) designed to amplify the CNV junction. The gel shows that clones that contain the CNV have the 280 bp sequence, while a 3D7 control does not.

Figure S5

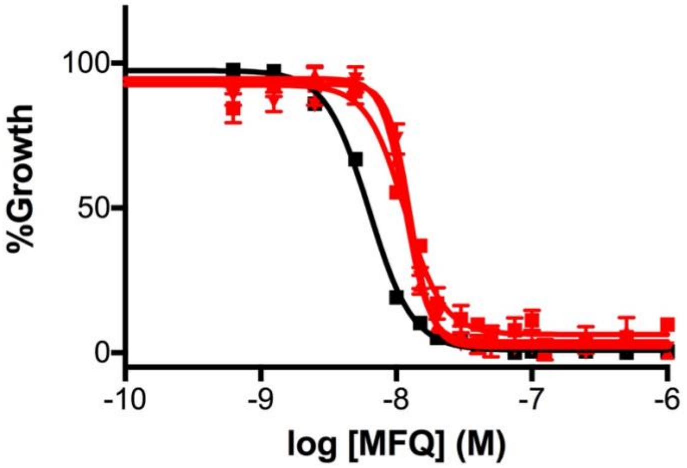

Fig. S5. Mefloquine cross-resistance in clones resistant to the compound MMV665789 (red lines; clones MALDA-MMV665789-C1, D8, and H7), which have an amplification surrounding *pfmdr1* compared to the sensitive 3D7 parent, which does not bear this amplification (black line; clone MALDA-3D7-Parent-5). MMV665789 resistant clones demonstrate an average resistance shift of 1.9x±0.1 fold. Dose-response experiments were done in triplicate with synchronous, young ring-stage cultures (1.0-1.2% starting parasitemia). Parasitemias (percentage of total erythrocytes infected with parasites) were measured approximately 70-80 hours post compound addition by nucleic acid staining of infected red blood cells with 0.8 mg/ml acridine orange in PBS. Inhibition data were fit to a sigmoidal dose-response curve using Graphpad Prism 5.0.

Figure S6

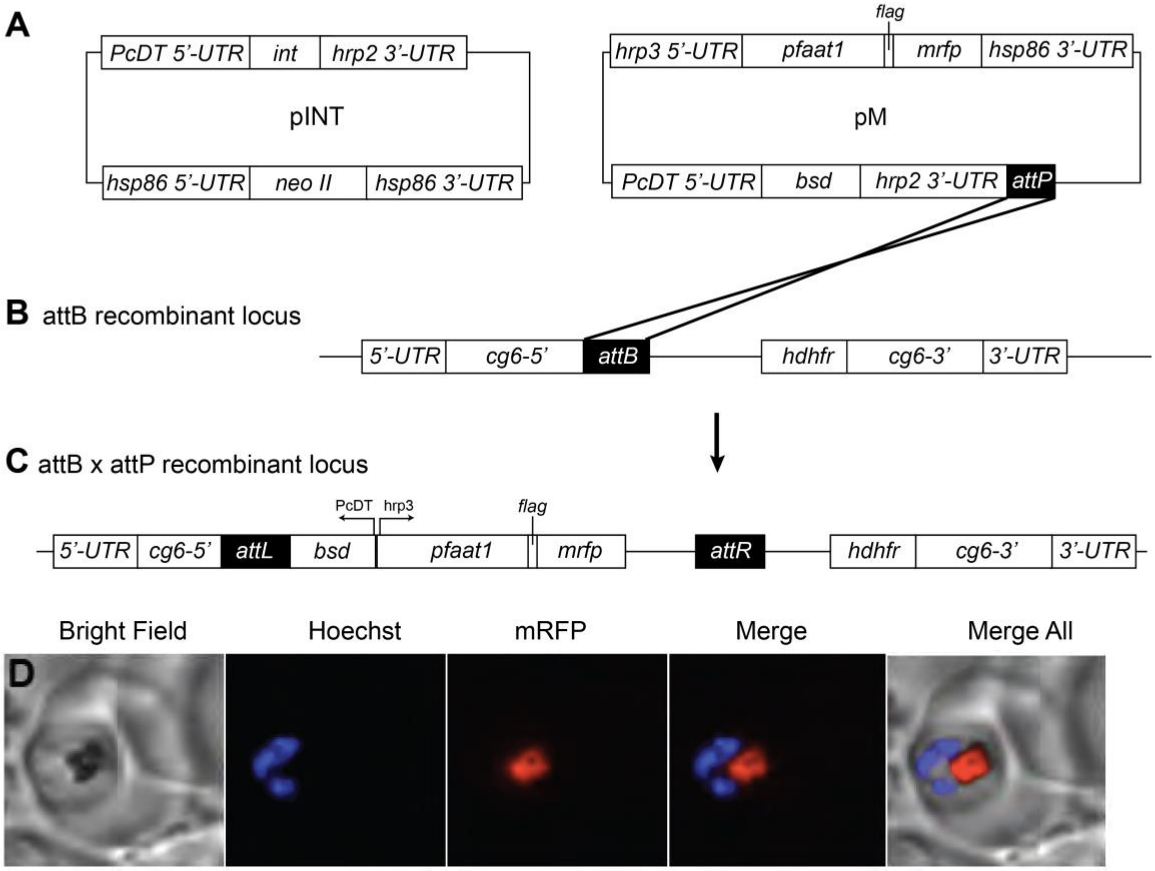

Fig. S6. PfAAT1 localizes to the digestive vacuole. An AAT1-mRFP fusion, under control of the *hrp3* promoter, was integrated in Dd2 using the mycobacteriophage Bxb1 integrase system[85]. (**A**) The pINT and pM plasmids were cotransfected into a previously generated $Dd2^{attB}$ line containing (**B**) an *attB* site at the cg6 locus. (**C**) *attB* × *attP* recombination catalyzed by the integrase, which is expressed from the pINT plasmid, results in integration of *pfaat1-mrfp* into the *attB* site. (D) Infected erythrocytes expressing the fluorescent fusion protein were stained with Hoechst and imaged live. UTR: untranslated region, *neo*: neomycin, *bsd*: blasticidin S-deaminase.

Figure S7

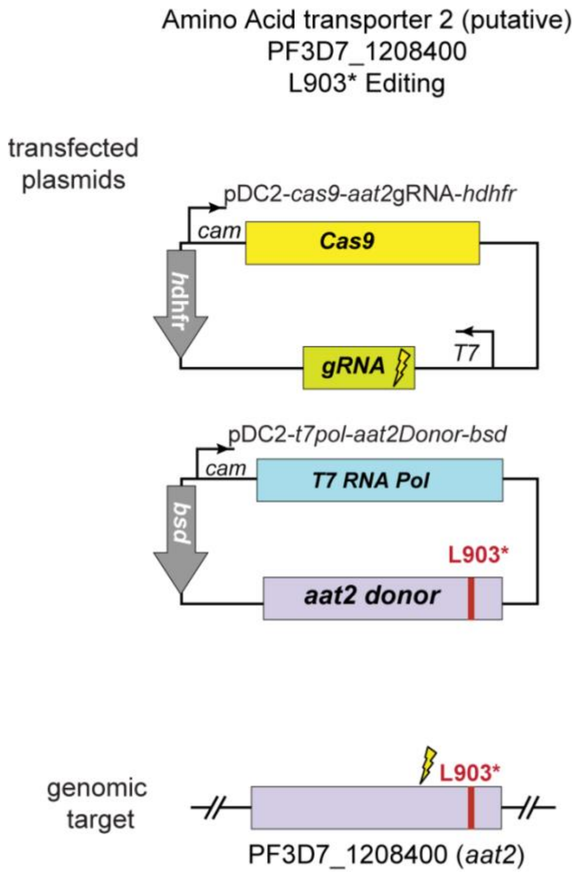

Fig. S7. Schematic indicating the cloning strategy used, via the two indicated plasmid constructs, to generate CRISPR/Cas9-derived *pfaat2* L903* mutant parasite clones. Selection markers for the two plasmids were blasticidin deaminase (BSD above) and human dihydrofolate reductase (hDHFR above). After transfection via electroporation, wild-type Dd2 parasites were treated with blasticidin (2.5 µg/ml) and WR99210 (2.5 nM) for 5 days, then cultured in the presence of 25 nM GNF179 until parasites were readily quantifiable via thin blood smear. CRISPR-Cas9 introduction of the L903 stop codon into Dd2 parasites showed that this conferred resistance to GNF179 relative to the Dd2 parent clone (EC_{50} = 0.115 µM; Dd2 parent 0.003 µM), although it was not as resistant as the original resistant line that also contained an acetyl CoA splice acceptor intronic mutation (EC_{50} = 5.7 µM, a 1,000–fold increase relative to 3D7 parasites).

Figure S8

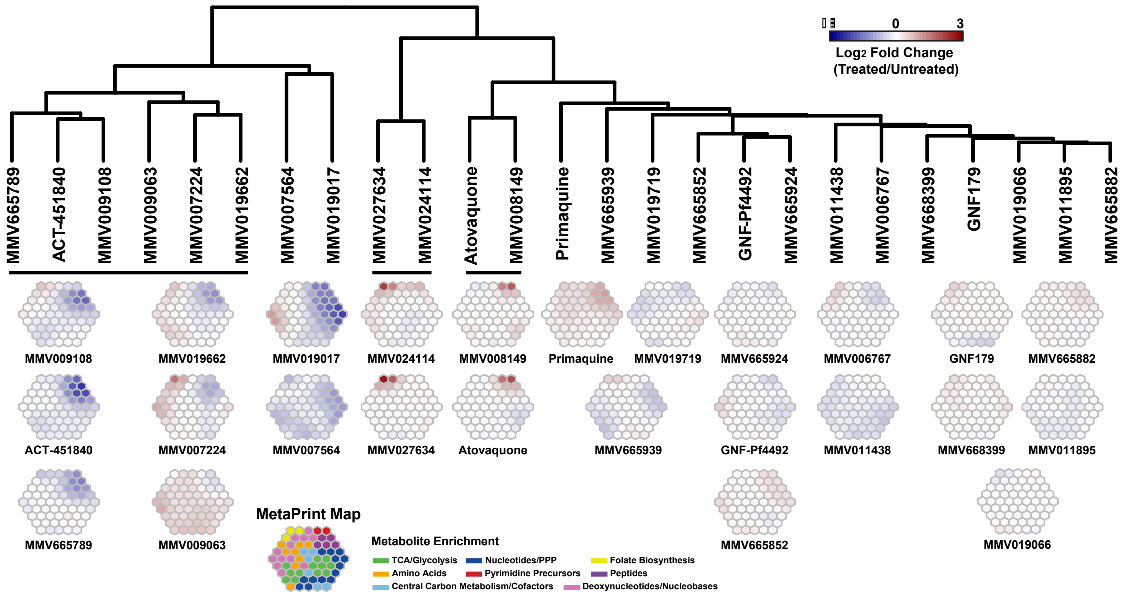

Fig. S8. Metabolomic fingerprint analysis of antimalarial compounds assayed in this study. The metabolic response of trophozoite stage *P. falciparum* parasites to treatment (10x IC_{50}) with each antimalarial compound was captured using metabolomic profiling. The average log2 fold change for each metabolite was determined following drug treatment compared to an untreated control (n=3) and the data were visualized using pre-determined MetaPrint self-organizing maps displayed as supra-hexagonal metabolic fingerprints[54]. Hex clusters were color-coded within the suprahexagon map based upon their metabolite similarity or association with eight generalized KEGG database metabolic pathways (inset) previously shown to be correlated across 20 antimalarial standards[54]. MetaPrints were clustered based on metabolite changes by Pearson-Ward distance and are displayed below their respective clusters. Bars above MetaPrint clusters represent correlated groups discussed in the main text. Overall, detected metabolic perturbations predict modes of action in accordance with the resistance mutations observed in resistant *P. falciparum* clones compared to the sensitive parent clone.

Figure S9

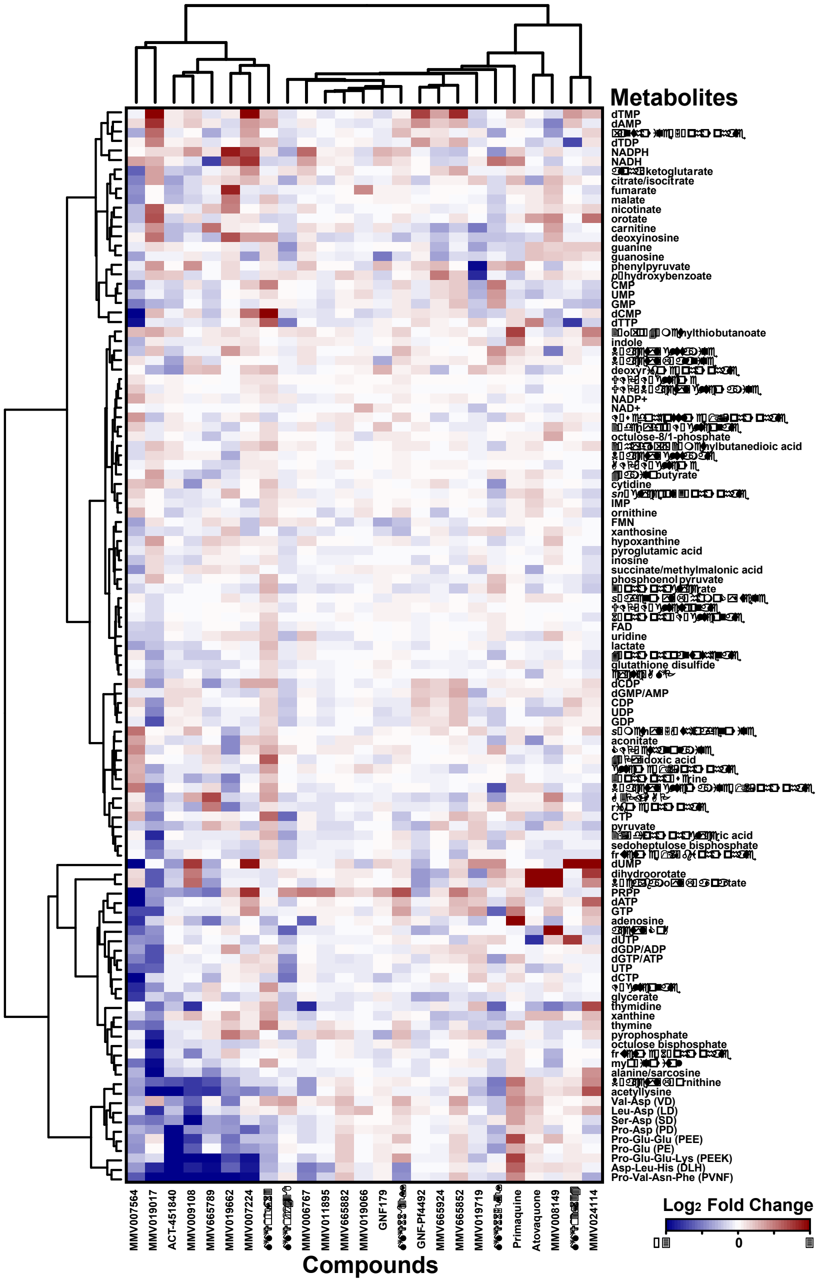

Fig S9. Metabolomic profiling of *P. falciparum* response to antimalarial compounds. Heatmap displaying average log2 fold change values of 113 metabolites measured by ultra-high performance liquid chromatography mass spectrometry (UHPLC-MS), relative to an untreated parasite control, for 25 antimalarial compounds. Changes in metabolites were clustered using Pearson-Ward distance. Compound names are displayed on the x-axis and measured metabolites are displayed on the y-axis. Fold changes are calculated for each metabolite from the average signal intensity of treated samples versus untreated parasites. All treatments were carried out at 10x IC_{50} for two and a half hours in triplicate technical replicate within a given trial. The full dataset is available in Table S16.

Figure S10

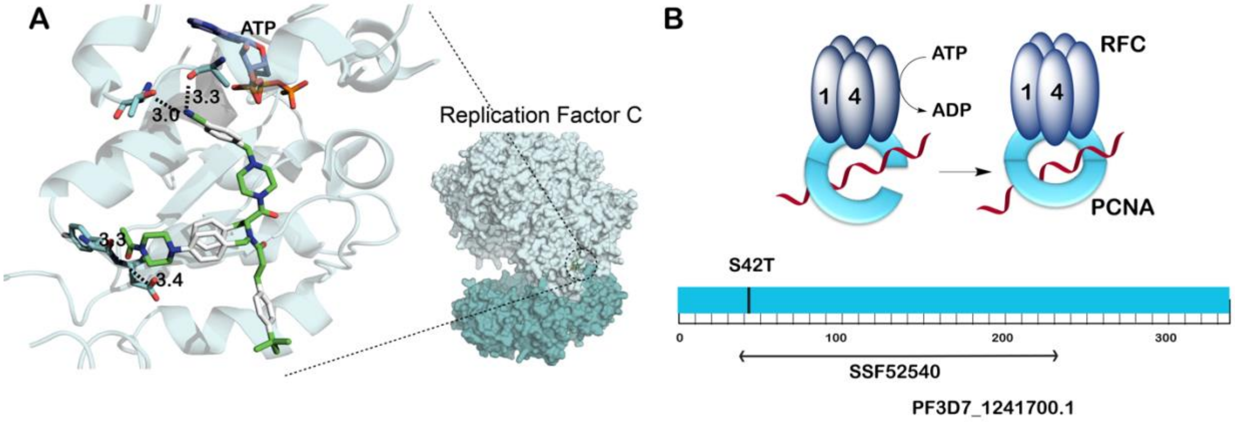

Fig. S10. S42T mutation in replication factor C subunit 4 may disrupt ATPγ ingress. (**A**) Left *S. cerevisiae* RFC (PDB: 1SXJ) was used as a template for docking to measure ligand affinity on an assembled complex rather than homology models of *P. falciparum RFC* subunits. Of the highest affinity conformations, ACT-451840 is shown nestled within the ATP binding pocket between homologous subunits 1 and 4 in *P. falciparum*. The flexibility of ACT-451840 allows for hydrogen-bond stabilization over a large area between subunits 1 and 4 of replication factor C, potentially disrupting ATPγ ingress. (**B**) Model for the function of replication factor C. Replication factor C is a five subunit complex that interacts with the clamp loader (PCNA) to initiate DNA replication by allowing DNA ingress in a ATP-dependent manner.

Table S7

	**Variants**	**Core Var.**	**Codon changing**	**No. of clones**	**Ref/Parent**
ACT-451840	19	7	6	4	7G8
Atovaquone	274	243	21	14	3D7[14]
BRD1095	8	7	6	7	Dd2
Cladosporin	613	22	5	3	Dd2[13]
GNF-Pf4492	6	6	5	3	Dd2[17]
GNF179	149	87	26	42	3D7, Dd2[63]
GNF707	30	8	6	3	Dd2
KAD452	8	5	3	1	Dd2[76]
MMV006767	13	12	4	5	3D7
MMV007224	25	14	5	3	3D7
MMV007564	6	5	3	9	3D7
MMV008149	10	8	3	6	Dd2
MMV009063	24	13	5	8	3D7
MMV009108	38	31	3	6	3D7
MMV011438	13	4	5	4	Dd2
MMV011895	58	16	5	4	3D7
MMV019017	28	10	6	4	3D7
MMV019066	11	11	3	3	3D7
MMV019662	92	78	6	15	3D7
MMV019719	6	5	3	6	3D7
MMV020746	13	13	5	3	3D7
MMV023367	6	6	4	5	Dd2
MMV024114	28	12	2	9	3D7
MMV026596	17	12	4	7	3D7
MMV027634	19	10	5	3	3D7
MMV028038	40	19	6	5	3D7
MMV029272	8	8	3	6	3D7
MMV665789	7	12	1	4	3D7
MMV665852	96	13	5	3	3D7
MMV665882	30	23	1	5	3D7
MMV665924	6	6	3	3	3D7
MMV665939	10	5	2	6	3D7
MMV668399	88	45	18	12	3D7
MMV673482	8	8	5	4	Dd2
NITD678	72	12	4	3	Dd2[76]
PBE	13	11	5	4	3D7[32]
Primaquine	53	30	4	7	Dd2
Parent lines	0	0	0	27	NA
Total	1945	784	206	262	

Table S7. Summary of small variants organized by compound used to evolve resistance. Multiple clones from each drug selection were typically sequenced along with at least one isogenic parent clone. In some cases, multiple clones from a single flask were sequenced. These typically yielded virtually identical data, so identical variants from the same round of selection were not counted twice. Core variants are those excluded from subtelomeric regions. Codon changing variants are nonsynonymous coding, splice site, start lost or gained, disruptive insertion or deletion indels and frameshift indels.

Table S8

ID	Name	Bgd No.	Result No.	Pct of bgd	Fold enrichment	Odds ratio	P-value	Benjamini	Bonferroni
GO:0042493	response to drug	39	8	20.5	12.56	13.94	5.78E-07	2.77E-05	2.77E-05
GO:0042221	response to chemical	61	8	13.1	8.03	8.87	1.16E-05	2.78E-04	5.56E-04
GO:0055085	transmembrane transport	87	9	10.3	6.34	7.06	1.85E-05	2.96E-04	8.87E-04
GO:0006810	transport	411	18	4.4	2.68	3.21	1.05E-04	1.03E-03	5.02E-03
GO:0051234	establishment of localization	412	18	4.4	2.68	3.2	1.08E-04	1.03E-03	5.17E-03
GO:0044765	single-organism transport	238	13	5.5	3.35	3.84	1.46E-04	1.07E-03	7.00E-03
GO:0051179	localization	429	18	4.2	2.57	3.07	1.77E-04	1.07E-03	8.52E-03
GO:1902578	single-organism localization	243	13	5.3	3.28	3.75	1.78E-04	1.07E-03	8.53E-03
GO:0006820	anion transport	48	5	10.4	6.38	6.76	1.47E-03	7.18E-03	7.07E-02
GO:0072348	sulfur compound transport	2	2	100	61.24	62.89	1.50E-03	7.18E-03	7.18E-02
GO:0006811	ion transport	104	7	6.7	4.12	4.44	1.86E-03	8.13E-03	8.94E-02
GO:0015893	drug transport	4	2	50	30.62	31.43	3.66E-03	1.35E-02	1.76E-01
GO:0006855	drug transmembrane transport	4	2	50	30.62	31.43	3.66E-03	1.35E-02	1.76E-01
GO:0044699	single-organism process	986	25	2.5	1.55	1.83	1.24E-02	3.99E-02	5.97E-01
GO:0015698	inorganic anion transport	28	3	10.7	6.56	6.79	1.30E-02	3.99E-02	6.22E-01
GO:0044763	single-organism cellular process	734	20	2.7	1.67	1.91	1.33E-02	3.99E-02	6.38E-01
GO:0071705	nitrogen compound transport	15	2	13.3	8.17	8.36	2.96E-02	4.14E-02	1.00E+00

Table S8. The 83 genes with nonsynonymous coding variants identified in this study are statistically enriched for genes with a *response to drug* gene ontology biological process designation (GO:0042493). Gene ontologies are from PlasmoDB release 27[84].

Table S9

Chr	CNVs	Possible target or resistance genes in amplified regions	Ave len.	Selection compounds
1	27	*pfmcp1* (PF3D7_0108400)<br>*pfmrp1* (PF3D7_0112200)<br>*pflss* (PF3D7_0107500)	26.7	GNF179, MMV008149,<br>MMV019662, MMV023367<br>MMV028038, MMV665882<br>none
2	1	None obvious	4	MMV665852
3	20	Putative formate-nitrite transporter (PF3D7_0316600)<br>*pfabcI3* (PF3D7_0319700)<br>*pfabcb4* (PF3D7_0302600)<br>*pfcarl* (PF3D7_0321900)	21.2	MMV006767, MMV008149<br>MMV019066, MMV023367<br>MMV024114, MMV029272<br>MMV665924, MMV665939
4	2	None obvious	11	MMV668399, none
5	37	*pfmdr1*(PF3D7_0523000)<br>*pfpi4k* (PF3D7_0509800)	13.7	BRD1095, MMV008149<br>MMV009063, MMV019017<br>MMV019066, MMV027634<br>MMV665789, MMV665882<br>MMV673482, none
7	13	None obvious	9.2	MMV006767, MMV007564<br>MMV008149, MMV019662<br>MMV665924, MMV668399
8	4	None obvious	11.5	MMV008149, MMV665789<br>MMV668399
9	2	None obvious	8	MMV008149<br>MMV023367
10	11	*pfap2tf-10* (PF3D7_1007700)<br>ADP/ATP transporter on adenylate translocase (PF3D7_1037300)<br>*pfact* (PF3D7_1036800)	11.6	GNF179, MMV008149<br>MMV011438, MMV019066<br>MMV026596, MMV668399<br>Primaquine
11	6	None obvious	11.2	BRD1095, GNF179<br>MMV007224, MMV008149
12	26	None obvious<br>*pfatp2* (PF3D7_1219600)<br>*pfaat2* (PF3D7_1208400)<br>putative mitochondrial carrier protein (PF3D7_1241600)	9.8	BRD1095, GNF179<br>MMV007224, MMV008149<br>MMV011438, MMV019662<br>MMV023367, MMV028038<br>MMV665852
13	9	*pfkrs1* (PF3D7_1350100)<br>None obvious	16	Cladosporin, MMV008149<br>MMV019066, MMV019662<br>MMV027634, MMV665852<br>MMV665924
14	1	None obvious	8	MMV008149
All	159			

Table S9. Summary of high quality copy number variants (CNVs) identified in this study. The Ave. Len. is the average size of the amplification defined by the number of genes contained within the region of amplification. Some CNVs covered several plausible candidate genes.

Table S14

Gene	Pos	Ref	Alt	Amino Acid Change	CNV	Mutation Call	Clones
PF3D7_0107500 (lipid sterol symporter)	304636	A	T	F1436I	No	Hom	1F4<br>1F9
	307789	G	A	S490L	No	Hom	2G6<br>2G9<br>3C3<br>3F10
	304938	G	A	I1382M	Yes	Het	2B6<br>2D6<br>2F6
	304945	A	G	I48L	Yes	Het	1C4
	307762	A	G	D292E	Yes	Het	3B6<br>G6

Table S14. Heterozygous single nucleotide variant (SNV) mutation calls (Het) are found in PF3D7_0107500 (putative lipid-sterol symporter) in clones resistant to MMV019662 that also have a CNV surrounding the gene. Clones that do not have a CNV surrounding PF3D7_0107500 have homozygous (Hom) SNVs in this gene.

Table S15

Chr	Gene ID	Gene Description	Nonsyn	Syn	Nonsyn:Syn ratio
1	PF3D7_0107500	Putative lipid-sterol symporter	201	163	1.2:1
3	**PF3D7_0319700**	**Putative ABC transporter**	**583**	**267**	**2.2:1**
	PF3D7_0321900	Cyclic amine resistance locus protein	255	148	1.7:1
4	**PF3D7_0417200**	**Bifunctional dihydrofolate reductase-thymidylate synthase (DHFR-TS)**	**16**	**7**	**2.3:1**
5	PF3D7_0523000	Multidrug resistance protein (MDR1)	105	103	1.0:1
6	**PF3D7_0613800**	**Transcription factor with AP2 domain(s) (ApiAP2)**	**905**	**356**	**2.5:1**
	PF3D7_0629500	Putative amino acid transporter	56	46	1.2:1
7	**PF3D7_0709000**	**chloroquine resistance transporter (CRT)**	**63**	**28**	**2.3:1**
	PF3D7_0709700	Putative lysophospholipase	22	34	0.7:1
9	PF3D7_0909700	Putative FHA domain protein	207	111	1.9:1
10	PF3D7_1007700	Transcription factor with AP2 domain(s) (ApiAP2)	242	132	1.8:1
	PF3D7_1036800	Putative acetyl-CoA transporter	58	63	0.9:1
11	PF3D7_1116700	cathepsin C, homolog,dipeptidyl peptidase 1+(DPAP1)	96	89	1.1:1
	**PF3D7_1147500**	**Putative farnesyltransferase beta subunit**	**272**	**126**	**2.2:1**
12	**PF3D7_1208400**	**Putative amino acid transporter**	**284**	**108**	**2.6:1**
	PF3D7_1211900	non-SERCA-type Ca2++-transporting P-ATPase (ATP4)	196	151	1.3:1
	PF3D7_1229100	ABC transporter, (CT family) (MRP2)	285	194	1.5:1
14	PF3D7_1447900	multidrug resistance protein 2+ (MDR2)	160	105	1.5:1
	PF3D7_1471200	inorganic anion exchanger, inorganic anion antiporter (SulP)	17	80	0.2:1

Table S15. Nonsynonymous (Nonsyn) to synonymous (Syn) allele counts and ratio for genes identified in this study as mediators of resistance or encoding potential antimalarial targets in a total of 3,247 samples, with data obtained from the Malaria Genomic Epidemiology Network (MalariaGEN) *Plasmodium falciparum* Community Project. Genes with a nonsynonymous to synonymous ratio greater than 2:1 are bolded.

Additional Data (Captions for Tables S1-S6, S10-S13, S16, found online)

Table S1. Detailed description of the 37 compounds with antimalarial activity used to create resistant *P. falciparum* clones.
This includes known compounds (e.g. atovaquone, cladosporin, and primaquine) in addition to novel compounds with antimalarial activity. The color coding system for each compound that is used in Figures 1 and 2 is displayed. The simplified molecular-input line-entry system (SMILES) specification and reference to previously published work describing the compound are shown.

Table S2. Compound concentration that results in half-maximal response (EC_{50}) for the 37 compounds used to generate resistance in three *P. falciparum* life cycle stages: asexual blood stage, liver stage, and the transmission stage (stage V gametocytes).
EC_{50} (concentration of a drug that gives half-maximal response) of the 37 compounds used in this study against the 3 *P. falciparum* life cycle stages that have been tested: asexual blood stage, liver stage, and the transmission stage (stage V gametocytes). The EC_{50} cutoffs used to define whether a compound has activity at a particular stage is shown.

Table S3. Detailed description of the 262 *P. falciparum* clone sequences analyzed in this study. The parent and average clone EC_{50} are shown, in addition to the EC_{50} fold shift (average clone EC_{50}/average parent EC_{50}). Average clone sequencing statistics and the SRA accession numbers are shown for each clone.

Table S4. Detailed information regarding the 1945 single nucleotide variants (SNVs) and insertions or deletions (indels) discovered in the set of 235 compound-resistant clones compared to their isogenic compound-sensitive parent clones.

Table S5. The 148 missense SNVs found in the set of 235 compound-resistant clones in comparison to their isogenic sensitive parent sequences. Enzyme Commission numbers (EC numbers) are given for each enzyme listed.

Table S6. The 83 genes that had missense SNVs in the set of 235 compound-resistant clones in comparison to their isogenic sensitive parent sequences.

Table S10. Summary of the 159 copy number variants (CNVs) classified as amplifications that were identified in this study. For each CNV, the genomic start and end positions are given. The possible target or resistance gene contained within that location is described. The p-value obtained from a t-test, Benjamini-Hochberg corrected p-values, and normalized average coverage over the CNV are shown.

Table S11. Detailed information about each of the copy number variants (CNVs) that were identified in this study.

Table S12. Summary of the 109 gene deletions identified in this study.

Table S13a. Antimalarial susceptibilities of a NF54 wild-type parasite line and an ACT-451840-selected NF54 parasite line that harbors the F806L mutation in PfMDR1. Table S13b. Antimalarial susceptibilities of 3D7 wild-type parasite line and MMV026596-selected clones. All data are expressed in nM and errors indicate the standard error of the mean based on 3 independent experiments.

Table S16. Log2 fold changes vs. untreated for 25 novel and well-established antimalarial compounds. Listed are the average log2 fold changes of 113 metabolites used to create heatmaps and MetaPrints of the parasite's response to 22 antimalarial compounds. All metabolite extractions are from parasites treated with 10x IC50 of each compound in three technical replicates in a single biological replicate except for Atovaquone (n = 4 biological replicates). Fold changes are calculated for each metabolite from the average signal intensity of treated sample (n = 3) versus untreated parasites (n = 3).

References and Notes

1. E. A. Ashley, M. Dhorda, R. M. Fairhurst, C. Amaratunga, P. Lim, S. Suon, S. Sreng, J. M. Anderson, S. Mao, B. Sam, C. Sopha, C. M. Chuor, C. Nguon, S. Sovannaroth, S. Pukrittayakamee, P. Jittamala, K. Chotivanich, K. Chutasmit, C. Suchatsoonthorn, R. Runcharoen, T. T. Hien, N. T. Thuy-Nhien, N. V. Thanh, N. H. Phu, Y. Htut, K.-T. Han, K. H. Aye, O. A. Mokuolu, R. R. Olaosebikan, O. O. Folaranmi, M. Mayxay, M. Khanthavong, B. Hongvanthong, P. N. Newton, M. A. Onyamboko, C. I. Fanello, A. K. Tshefu, N. Mishra, N. Valecha, A. P. Phyo, F. Nosten, P. Yi, R. Tripura, S. Borrmann, M. Bashraheil, J. Peshu, M. A. Faiz, A. Ghose, M. A. Hossain, R. Samad, M. R. Rahman, M. M. Hasan, A. Islam, O. Miotto, R. Amato, B. MacInnis, J. Stalker, D. P. Kwiatkowski, Z. Bozdech, A. Jeeyapant, P. Y. Cheah, T. Sakulthaew, J. Chalk, B. Intharabut, K. Silamut, S. J. Lee, B. Vihokhern, C. Kunasol, M. Imwong, J. Tarning, W. J. Taylor, S. Yeung, C. J. Woodrow, J. A. Flegg, D. Das, J. Smith, M. Venkatesan, C. V. Plowe, K. Stepniewska, P. J. Guerin, A. M. Dondorp, N. P. Day, N. J. White; Tracking Resistance to Artemisinin Collaboration (TRAC), Spread of artemisinin resistance in *Plasmodium falciparum* malaria. *N. Engl. J. Med.* **371**, 411–423 (2014). doi:10.1056/NEJMoa1314981 Medline

2. F. Ariey, B. Witkowski, C. Amaratunga, J. Beghain, A.-C. Langlois, N. Khim, S. Kim, V. Duru, C. Bouchier, L. Ma, P. Lim, R. Leang, S. Duong, S. Sreng, S. Suon, C. M. Chuor, D. M. Bout, S. Ménard, W. O. Rogers, B. Genton, T. Fandeur, O. Miotto, P. Ringwald, J. Le Bras, A. Berry, J.-C. Barale, R. M. Fairhurst, F. Benoit-Vical, O. Mercereau-Puijalon, D. Ménard, A molecular marker of artemisinin-resistant *Plasmodium falciparum* malaria. *Nature* **505**, 50–55 (2014). doi:10.1038/nature12876 Medline

3. E. L. Flannery, D. A. Fidock, E. A. Winzeler, Using genetic methods to define the targets of compounds with antimalarial activity. *J. Med. Chem.* **56**, 7761–7771 (2013). doi:10.1021/jm400325j Medline

4. T. Spangenberg, J. N. Burrows, P. Kowalczyk, S. McDonald, T. N. C. Wells, P. Willis, The open access malaria box: A drug discovery catalyst for neglected diseases. *PLOS ONE* **8**, e62906 (2013). doi:10.1371/journal.pone.0062906 Medline

5. F.-J. Gamo, L. M. Sanz, J. Vidal, C. de Cozar, E. Alvarez, J.-L. Lavandera, D. E. Vanderwall, D. V. S. Green, V. Kumar, S. Hasan, J. R. Brown, C. E. Peishoff, L. R. Cardon, J. F. Garcia-Bustos, Thousands of chemical starting points for antimalarial lead identification. *Nature* **465**, 305–310 (2010). doi:10.1038/nature09107 Medline

6. W. A. Guiguemde, A. A. Shelat, D. Bouck, S. Duffy, G. J. Crowther, P. H. Davis, D. C. Smithson, M. Connelly, J. Clark, F. Zhu, M. B. Jiménez-Díaz, M. S. Martinez, E. B. Wilson, A. K. Tripathi, J. Gut, E. R. Sharlow, I. Bathurst, F. El Mazouni, J. W. Fowble, I. Forquer, P. L. McGinley, S. Castro, I. Angulo-Barturen, S. Ferrer, P. J. Rosenthal, J. L. Derisi, D. J. Sullivan, J. S. Lazo, D. S. Roos, M. K. Riscoe, M. A. Phillips, P. K. Rathod, W. C. Van Voorhis, V. M. Avery, R. K. Guy,

Chemical genetics of *Plasmodium falciparum*. *Nature* **465**, 311–315 (2010). doi:10.1038/nature09099 Medline

7. D. Plouffe, A. Brinker, C. McNamara, K. Henson, N. Kato, K. Kuhen, A. Nagle, F. Adrián, J. T. Matzen, P. Anderson, T. G. Nam, N. S. Gray, A. Chatterjee, J. Janes, S. F. Yan, R. Trager, J. S. Caldwell, P. G. Schultz, Y. Zhou, E. A. Winzeler, In silico activity profiling reveals the mechanism of action of antimalarials discovered in a high-throughput screen. *Proc. Natl. Acad. Sci. U.S.A.* **105**, 9059–9064 (2008). doi:10.1073/pnas.0802982105 Medline
8. M. Rottmann, C. McNamara, B. K. S. Yeung, M. C. S. Lee, B. Zou, B. Russell, P. Seitz, D. M. Plouffe, N. V. Dharia, J. Tan, S. B. Cohen, K. R. Spencer, G. E. González-Páez, S. B. Lakshminarayana, A. Goh, R. Suwanarusk, T. Jegla, E. K. Schmitt, H.-P. Beck, R. Brun, F. Nosten, L. Renia, V. Dartois, T. H. Keller, D. A. Fidock, E. A. Winzeler, T. T. Diagana, Spiroindolones, a potent compound class for the treatment of malaria. *Science* **329**, 1175–1180 (2010). doi:10.1126/science.1193225 Medline
9. S. Meister, D. M. Plouffe, K. L. Kuhen, G. M. C. Bonamy, T. Wu, S. W. Barnes, S. E. Bopp, R. Borboa, A. T. Bright, J. Che, S. Cohen, N. V. Dharia, K. Gagaring, M. Gettayacamin, P. Gordon, T. Groessl, N. Kato, M. C. S. Lee, C. W. McNamara, D. A. Fidock, A. Nagle, T. G. Nam, W. Richmond, J. Roland, M. Rottmann, B. Zhou, P. Froissard, R. J. Glynne, D. Mazier, J. Sattabongkot, P. G. Schultz, T. Tuntland, J. R. Walker, Y. Zhou, A. Chatterjee, T. T. Diagana, E. A. Winzeler, Imaging of *Plasmodium* liver stages to drive next-generation antimalarial drug discovery. *Science* **334**, 1372–1377 (2011). doi:10.1126/science.1211936 Medline
10. K. L. Kuhen, A. K. Chatterjee, M. Rottmann, K. Gagaring, R. Borboa, J. Buenviaje, Z. Chen, C. Francek, T. Wu, A. Nagle, S. W. Barnes, D. Plouffe, M. C. S. Lee, D. A. Fidock, W. Graumans, M. van de Vegte-Bolmer, G. J. van Gemert, G. Wirjanata, B. Sebayang, J. Marfurt, B. Russell, R. Suwanarusk, R. N. Price, F. Nosten, A. Tungtaeng, M. Gettayacamin, J. Sattabongkot, J. Taylor, J. R. Walker, D. Tully, K. P. Patra, E. L. Flannery, J. M. Vinetz, L. Renia, R. W. Sauerwein, E. A. Winzeler, R. J. Glynne, T. T. Diagana, KAF156 is an antimalarial clinical candidate with potential for use in prophylaxis, treatment, and prevention of disease transmission. *Antimicrob. Agents Chemother.* **58**, 5060–5067 (2014). doi:10.1128/AAC.02727-13 Medline
11. N. J. White, S. Pukrittayakamee, A. P. Phyo, R. Rueangweerayut, F. Nosten, P. Jittamala, A. Jeeyapant, J. P. Jain, G. Lefèvre, R. Li, B. Magnusson, T. T. Diagana, F. J. Leong, Spiroindolone KAE609 for falciparum and vivax malaria. *N. Engl. J. Med.* **371**, 403–410 (2014). doi:10.1056/NEJMoa1315860 Medline
12. V. C. Corey, A. K. Lukens, E. S. Istvan, M. C. S. Lee, V. Franco, P. Magistrado, O. Coburn-Flynn, T. Sakata-Kato, O. Fuchs, N. F. Gnädig, G. Goldgof, M. Linares, M. G. Gomez-Lorenzo, C. De Cózar, M. J. Lafuente-Monasterio, S. Prats, S. Meister, O. Tanaseichuk, M. Wree, Y. Zhou, P. A. Willis, F.-J. Gamo, D. E. Goldberg, D. A. Fidock, D. F. Wirth, E. A. Winzeler, A broad analysis of resistance development in the malaria parasite. *Nat. Commun.* **7**, 11901 (2016). doi:10.1038/ncomms11901 Medline

13. D. Hoepfner, C. W. McNamara, C. S. Lim, C. Studer, R. Riedl, T. Aust, S. L. McCormack, D. M. Plouffe, S. Meister, S. Schuierer, U. Plikat, N. Hartmann, F. Staedtler, S. Cotesta, E. K. Schmitt, F. Petersen, F. Supek, R. J. Glynne, J. A. Tallarico, J. A. Porter, M. C. Fishman, C. Bodenreider, T. T. Diagana, N. R. Movva, E. A. Winzeler, Selective and specific inhibition of the plasmodium falciparum lysyl-tRNA synthetase by the fungal secondary metabolite cladosporin. *Cell Host Microbe* **11**, 654–663 (2012). doi:10.1016/j.chom.2012.04.015 Medline

14. S. E. Bopp, M. J. Manary, A. T. Bright, G. L. Johnston, N. V. Dharia, F. L. Luna, S. McCormack, D. Plouffe, C. W. McNamara, J. R. Walker, D. A. Fidock, E. L. Denchi, E. A. Winzeler, Mitotic evolution of *Plasmodium falciparum* shows a stable core genome but recombination in antigen families. *PLOS Genet.* **9**, e1003293 (2013). doi:10.1371/journal.pgen.1003293 Medline

15. M. Y. Lim, G. LaMonte, M. C. S. Lee, C. Reimer, B. H. Tan, V. Corey, B. F. Tjahjadi, A. Chua, M. Nachon, R. Wintjens, P. Gedeck, B. Malleret, L. Renia, G. M. C. Bonamy, P. C.-L. Ho, B. K. S. Yeung, E. D. Chow, L. Lim, D. A. Fidock, T. T. Diagana, E. A. Winzeler, P. Bifani, UDP-galactose and acetyl-CoA transporters as *Plasmodium* multidrug resistance genes. *Nat. Microbiol.* **1**, 16166 (2016). doi:10.1038/nmicrobiol.2016.166 Medline

16. N. V. Dharia, D. Plouffe, S. E. R. Bopp, G. E. González-Páez, C. Lucas, C. Salas, V. Soberon, B. Bursulaya, T. J. Kochel, D. J. Bacon, E. A. Winzeler, Genome scanning of Amazonian *Plasmodium falciparum* shows subtelomeric instability and clindamycin-resistant parasites. *Genome Res.* **20**, 1534–1544 (2010). doi:10.1101/gr.105163.110 Medline

17. E. L. Flannery, C. W. McNamara, S. W. Kim, T. S. Kato, F. Li, C. H. Teng, K. Gagaring, M. J. Manary, R. Barboa, S. Meister, K. Kuhen, J. M. Vinetz, A. K. Chatterjee, E. A. Winzeler, Mutations in the P-type cation-transporter ATPase 4, PfATP4, mediate resistance to both aminopyrazole and spiroindolone antimalarials. *ACS Chem. Biol.* **10**, 413–420 (2015). doi:10.1021/cb500616x Medline

18. S. Nair, D. Nash, D. Sudimack, A. Jaidee, M. Barends, A.-C. Uhlemann, S. Krishna, F. Nosten, T. J. C. Anderson, Recurrent gene amplification and soft selective sweeps during evolution of multidrug resistance in malaria parasites. *Mol. Biol. Evol.* **24**, 562–573 (2007). doi:10.1093/molbev/msl185 Medline

19. S. Nair, B. Miller, M. Barends, A. Jaidee, J. Patel, M. Mayxay, P. Newton, F. Nosten, M. T. Ferdig, T. J. C. Anderson, Adaptive copy number evolution in malaria parasites. *PLOS Genet.* **4**, e1000243 (2008). doi:10.1371/journal.pgen.1000243 Medline

20. J. L. Guler, D. L. Freeman, V. Ahyong, R. Patrapuvich, J. White, R. Gujjar, M. A. Phillips, J. DeRisi, P. K. Rathod, Asexual populations of the human malaria parasite, Plasmodium falciparum, use a two-step genomic strategy to acquire accurate, beneficial DNA amplifications. *PLOS Pathog.* **9**, e1003375 (2013). doi:10.1371/journal.ppat.1003375 Medline

21. B. Blasco, D. Leroy, D. A. Fidock, Antimalarial drug resistance: Linking *Plasmodium falciparum* parasite biology to the clinic. *Nat. Med.* **23**, 917–928 (2017). doi:10.1038/nm.4381 Medline

22. S. M. Teo, Y. Pawitan, C. S. Ku, K. S. Chia, A. Salim, Statistical challenges associated with detecting copy number variations with next-generation sequencing. *Bioinformatics* **28**, 2711–2718 (2012). doi:10.1093/bioinformatics/bts535 Medline

23. M. J. Gardner, N. Hall, E. Fung, O. White, M. Berriman, R. W. Hyman, J. M. Carlton, A. Pain, K. E. Nelson, S. Bowman, I. T. Paulsen, K. James, J. A. Eisen, K. Rutherford, S. L. Salzberg, A. Craig, S. Kyes, M.-S. Chan, V. Nene, S. J. Shallom, B. Suh, J. Peterson, S. Angiuoli, M. Pertea, J. Allen, J. Selengut, D. Haft, M. W. Mather, A. B. Vaidya, D. M. A. Martin, A. H. Fairlamb, M. J. Fraunholz, D. S. Roos, S. A. Ralph, G. I. McFadden, L. M. Cummings, G. M. Subramanian, C. Mungall, J. C. Venter, D. J. Carucci, S. L. Hoffman, C. Newbold, R. W. Davis, C. M. Fraser, B. Barrell, Genome sequence of the human malaria parasite *Plasmodium falciparum*. *Nature* **419**, 498–511 (2002). doi:10.1038/nature01097 Medline

24. J. T. Robinson, H. Thorvaldsdóttir, W. Winckler, M. Guttman, E. S. Lander, G. Getz, J. P. Mesirov, Integrative genomics viewer. *Nat. Biotechnol.* **29**, 24–26 (2011). doi:10.1038/nbt.1754 Medline

25. H. Thorvaldsdóttir, J. T. Robinson, J. P. Mesirov, Integrative Genomics Viewer (IGV): High-performance genomics data visualization and exploration. *Brief. Bioinform.* **14**, 178–192 (2013). doi:10.1093/bib/bbs017 Medline

26. D. Menard, E. R. Chan, C. Benedet, A. Ratsimbasoa, S. Kim, P. Chim, C. Do, B. Witkowski, R. Durand, M. Thellier, C. Severini, E. Legrand, L. Musset, B. Y. M. Nour, O. Mercereau-Puijalon, D. Serre, P. A. Zimmerman, Whole genome sequencing of field isolates reveals a common duplication of the Duffy binding protein gene in Malagasy *Plasmodium vivax* strains. *PLOS Negl. Trop. Dis.* **7**, e2489 (2013). doi:10.1371/journal.pntd.0002489 Medline

27. C. L. Ng, G. Siciliano, M. C. S. Lee, M. J. de Almeida, V. C. Corey, S. E. Bopp, L. Bertuccini, S. Wittlin, R. G. Kasdin, A. Le Bihan, M. Clozel, E. A. Winzeler, P. Alano, D. A. Fidock, CRISPR-Cas9-modified *pfmdr1* protects *Plasmodium falciparum* asexual blood stages and gametocytes against a class of piperazine-containing compounds but potentiates artemisinin-based combination therapy partner drugs. *Mol. Microbiol.* **101**, 381–393 (2016). doi:10.1111/mmi.13397 Medline

28. R. A. Kavishe, J. M. W. van den Heuvel, M. van de Vegte-Bolmer, A. J. F. Luty, F. G. M. Russel, J. B. Koenderink, Localization of the ATP-binding cassette (ABC) transport proteins PfMRP1, PfMRP2, and PfMDR5 at the *Plasmodium falciparum* plasma membrane. *Malar. J.* **8**, 205 (2009). doi:10.1186/1475-2875-8-205 Medline

29. S. Mok, K.-Y. Liong, E.-H. Lim, X. Huang, L. Zhu, P. R. Preiser, Z. Bozdech, Structural polymorphism in the promoter of *pfmrp2* confers *Plasmodium*

falciparum tolerance to quinoline drugs. *Mol. Microbiol.* **91**, 918–934 (2014). doi:10.1111/mmi.12505 Medline

30. B. Gasnier, The SLC32 transporter, a key protein for the synaptic release of inhibitory amino acids. *Pflugers Arch.* **447**, 756–759 (2004). doi:10.1007/s00424-003-1091-2 Medline

31. Z. Wang, M. Cabrera, J. Yang, L. Yuan, B. Gupta, X. Liang, K. Kemirembe, S. Shrestha, A. Brashear, X. Li, S. F. Porcella, J. Miao, Z. Yang, X. Z. Su, L. Cui, Genome-wide association analysis identifies genetic loci associated with resistance to multiple antimalarials in *Plasmodium falciparum* from China-Myanmar border. *Sci. Rep.* **6**, 33891 (2016). doi:10.1038/srep33891 Medline

32. E. S. Istvan, J. P. Mallari, V. C. Corey, N. V. Dharia, G. R. Marshall, E. A. Winzeler, D. E. Goldberg, Esterase mutation is a mechanism of resistance to antimalarial compounds. *Nat. Commun.* **8**, 14240 (2017). doi:10.1038/ncomms14240 Medline

33. S. Paul, W. S. Moye-Rowley, Multidrug resistance in fungi: Regulation of transporter-encoding gene expression. *Front. Physiol.* **5**, 143 (2014). doi:10.3389/fphys.2014.00143 Medline

34. J. L. Riechmann, E. M. Meyerowitz, The AP2/EREBP family of plant transcription factors. *Biol. Chem.* **379**, 633–646 (1998). Medline

35. T. L. Campbell, E. K. De Silva, K. L. Olszewski, O. Elemento, M. Llinás, Identification and genome-wide prediction of DNA binding specificities for the ApiAP2 family of regulators from the malaria parasite. *PLOS Pathog.* **6**, e1001165 (2010). doi:10.1371/journal.ppat.1001165 Medline

36. K. Modrzynska, C. Pfander, L. Chappell, L. Yu, C. Suarez, K. Dundas, A. R. Gomes, D. Goulding, J. C. Rayner, J. Choudhary, O. Billker, A knockout screen of ApiAP2 genes reveals networks of interacting transcriptional regulators controlling the *Plasmodium* life cycle. *Cell Host Microbe* **21**, 11–22 (2017). doi:10.1016/j.chom.2016.12.003 Medline

37. J. M. Santos, G. Josling, P. Ross, P. Joshi, L. Orchard, T. Campbell, A. Schieler, I. M. Cristea, M. Llinás, Red blood cell invasion by the malaria parasite is coordinated by the PfAP2-I transcription factor. *Cell Host Microbe* **21**, 731–741.e10 (2017). doi:10.1016/j.chom.2017.05.006 Medline

38. J. P. Wendler, J. Okombo, R. Amato, O. Miotto, S. M. Kiara, L. Mwai, L. Pole, J. O'Brien, M. Manske, D. Alcock, E. Drury, M. Sanders, S. O. Oyola, C. Malangone, D. Jyothi, A. Miles, K. A. Rockett, B. L. MacInnis, K. Marsh, P. Bejon, A. Nzila, D. P. Kwiatkowski, A genome wide association study of *Plasmodium falciparum* susceptibility to 22 antimalarial drugs in Kenya. *PLOS ONE* **9**, e96486 (2014). doi:10.1371/journal.pone.0096486 Medline

39. A. W. Almawi, L. A. Matthews, A. Guarne, FHA domains: Phosphopeptide binding and beyond. *Prog. Biophys. Mol. Biol.* **127**, 105–110 (2017). Medline

40. N. Kato, E. Comer, T. Sakata-Kato, A. Sharma, M. Sharma, M. Maetani, J. Bastien, N. M. Brancucci, J. A. Bittker, V. Corey, D. Clarke, E. R. Derbyshire, G. L. Dornan, S. Duffy, S. Eckley, M. A. Itoe, K. M. J. Koolen, T. A. Lewis, P. S. Lui,

A. K. Lukens, E. Lund, S. March, E. Meibalan, B. C. Meier, J. A. McPhail, B. Mitasev, E. L. Moss, M. Sayes, Y. Van Gessel, M. J. Wawer, T. Yoshinaga, A.-M. Zeeman, V. M. Avery, S. N. Bhatia, J. E. Burke, F. Catteruccia, J. C. Clardy, P. A. Clemons, K. J. Dechering, J. R. Duvall, M. A. Foley, F. Gusovsky, C. H. M. Kocken, M. Marti, M. L. Morningstar, B. Munoz, D. E. Neafsey, A. Sharma, E. A. Winzeler, D. F. Wirth, C. A. Scherer, S. L. Schreiber, Diversity-oriented synthesis yields novel multistage antimalarial inhibitors. *Nature* **538**, 344–349 (2016). doi:10.1038/nature19804 Medline

41. J. Yuvaniyama, P. Chitnumsub, S. Kamchonwongpaisan, J. Vanichtanankul, W. Sirawaraporn, P. Taylor, M. D. Walkinshaw, Y. Yuthavong, Insights into antifolate resistance from malarial DHFR-TS structures. *Nat. Struct. Biol.* **10**, 357–365 (2003). doi:10.1038/nsb921 Medline

42. R. T. Eastman, J. White, O. Hucke, K. Yokoyama, C. L. M. J. Verlinde, M. A. Hast, L. S. Beese, M. H. Gelb, P. K. Rathod, W. C. Van Voorhis, Resistance mutations at the lipid substrate binding site of *Plasmodium falciparum* protein farnesyltransferase. *Mol. Biochem. Parasitol.* **152**, 66–71 (2007). doi:10.1016/j.molbiopara.2006.11.012 Medline

43. F. Wang, P. Krai, E. Deu, B. Bibb, C. Lauritzen, J. Pedersen, M. Bogyo, M. Klemba, Biochemical characterization of *Plasmodium falciparum* dipeptidyl aminopeptidase 1. *Mol. Biochem. Parasitol.* **175**, 10–20 (2011). doi:10.1016/j.molbiopara.2010.08.004 Medline

44. M. Klemba, I. Gluzman, D. E. Goldberg, A *Plasmodium falciparum* dipeptidyl aminopeptidase I participates in vacuolar hemoglobin degradation. *J. Biol. Chem.* **279**, 43000–43007 (2004). doi:10.1074/jbc.M408123200 Medline

45. S. Kenthirapalan, A. P. Waters, K. Matuschewski, T. W. Kooij, Functional profiles of orphan membrane transporters in the life cycle of the malaria parasite. *Nat. Commun.* **7**, 10519 (2016). doi:10.1038/ncomms10519 Medline

46. C. C. Paulusma, R. P. Oude Elferink, The type 4 subfamily of P-type ATPases, putative aminophospholipid translocases with a role in human disease. *Biochim. Biophys. Acta* **1741**, 11–24 (2005). doi:10.1016/j.bbadis.2005.04.006 Medline

47. H. P. Schweizer, Triclosan: A widely used biocide and its link to antibiotics. *FEMS Microbiol. Lett.* **202**, 1–7 (2001). doi:10.1111/j.1574-6968.2001.tb10772.x Medline

48. M. Yu, T. R. S. Kumar, L. J. Nkrumah, A. Coppi, S. Retzlaff, C. D. Li, B. J. Kelly, P. A. Moura, V. Lakshmanan, J. S. Freundlich, J.-C. Valderramos, C. Vilcheze, M. Siedner, J. H.-C. Tsai, B. Falkard, A. B. Sidhu, L. A. Purcell, P. Gratraud, L. Kremer, A. P. Waters, G. Schiehser, D. P. Jacobus, C. J. Janse, A. Ager, W. R. Jacobs Jr., J. C. Sacchettini, V. Heussler, P. Sinnis, D. A. Fidock, The fatty acid biosynthesis enzyme FabI plays a key role in the development of liver-stage malarial parasites. *Cell Host Microbe* **4**, 567–578 (2008). doi:10.1016/j.chom.2008.11.001 Medline

49. C. Alvarez-Ortega, J. Olivares, J. L. Martínez, RND multidrug efflux pumps: What are they good for? *Front. Microbiol.* **4**, 7 (2013). doi:10.3389/fmicb.2013.00007 Medline

50. S. Coyne, N. Rosenfeld, T. Lambert, P. Courvalin, B. Périchon, Overexpression of resistance-nodulation-cell division pump AdeFGH confers multidrug resistance in *Acinetobacter baumannii*. *Antimicrob. Agents Chemother.* **54**, 4389–4393 (2010). doi:10.1128/AAC.00155-10 Medline

51. S. L. Alper, A. K. Sharma, The SLC26 gene family of anion transporters and channels. *Mol. Aspects Med.* **34**, 494–515 (2013). doi:10.1016/j.mam.2012.07.009 Medline

52. R. I. Henry, R. E. Martin, S. M. Howitt, K. Kirk, Localisation of a candidate anion transporter to the surface of the malaria parasite. *Biochem. Biophys. Res. Commun.* **363**, 288–291 (2007). doi:10.1016/j.bbrc.2007.08.116 Medline

53. E. R. Geertsma, Y.-N. Chang, F. R. Shaik, Y. Neldner, E. Pardon, J. Steyaert, R. Dutzler, Structure of a prokaryotic fumarate transporter reveals the architecture of the SLC26 family. *Nat. Struct. Mol. Biol.* **22**, 803–808 (2015). doi:10.1038/nsmb.3091 Medline

54. E. L. Allman, H. J. Painter, J. Samra, M. Carrasquilla, M. Llinás, Metabolomic profiling of the malaria box reveals antimalarial target pathways. *Antimicrob. Agents Chemother.* **60**, 6635–6649 (2016). doi:10.1128/AAC.01224-16 Medline

55. J. Mu, R. A. Myers, H. Jiang, S. Liu, S. Ricklefs, M. Waisberg, K. Chotivanich, P. Wilairatana, S. Krudsood, N. J. White, R. Udomsangpetch, L. Cui, M. Ho, F. Ou, H. Li, J. Song, G. Li, X. Wang, S. Seila, S. Sokunthea, D. Socheat, D. E. Sturdevant, S. F. Porcella, R. M. Fairhurst, T. E. Wellems, P. Awadalla, X. Z. Su, *Plasmodium falciparum* genome-wide scans for positive selection, recombination hot spots and resistance to antimalarial drugs. *Nat. Genet.* **42**, 268–271 (2010). doi:10.1038/ng.528 Medline

56. S. Borrmann, J. Straimer, L. Mwai, A. Abdi, A. Rippert, J. Okombo, S. Muriithi, P. Sasi, M. M. Kortok, B. Lowe, S. Campino, S. Assefa, S. Auburn, M. Manske, G. Maslen, N. Peshu, D. P. Kwiatkowski, K. Marsh, A. Nzila, T. G. Clark, Genome-wide screen identifies new candidate genes associated with artemisinin susceptibility in *Plasmodium falciparum* in Kenya. *Sci. Rep.* **3**, 3318 (2013). doi:10.1038/srep03318 Medline

57. M. B. Jiménez-Díaz, D. Ebert, Y. Salinas, A. Pradhan, A. M. Lehane, M.-E. Myrand-Lapierre, K. G. O'Loughlin, D. M. Shackleford, M. Justino de Almeida, A. K. Carrillo, J. A. Clark, A. S. M. Dennis, J. Diep, X. Deng, S. Duffy, A. N. Endsley, G. Fedewa, W. A. Guiguemde, M. G. Gómez, G. Holbrook, J. Horst, C. C. Kim, J. Liu, M. C. S. Lee, A. Matheny, M. S. Martínez, G. Miller, A. Rodríguez-Alejandre, L. Sanz, M. Sigal, N. J. Spillman, P. D. Stein, Z. Wang, F. Zhu, D. Waterson, S. Knapp, A. Shelat, V. M. Avery, D. A. Fidock, F.-J. Gamo, S. A. Charman, J. C. Mirsalis, H. Ma, S. Ferrer, K. Kirk, I. Angulo-Barturen, D. E. Kyle, J. L. DeRisi, D. M. Floyd, R. K. Guy, (+)-SJ733, a clinical candidate for malaria that acts through ATP4 to induce rapid host-mediated clearance of

Plasmodium. *Proc. Natl. Acad. Sci. U.S.A.* **111**, E5455–E5462 (2014). doi:10.1073/pnas.1414221111 Medline

58. C. W. McNamara, M. C. S. Lee, C. S. Lim, S. H. Lim, J. Roland, O. Simon, B. K. Yeung, A. K. Chatterjee, S. L. McCormack, M. J. Manary, A. M. Zeeman, K. J. Dechering, T. S. Kumar, P. P. Henrich, K. Gagaring, M. Ibanez, N. Kato, K. L. Kuhen, C. Fischli, A. Nagle, M. Rottmann, D. M. Plouffe, B. Bursulaya, S. Meister, L. Rameh, J. Trappe, D. Haasen, M. Timmerman, R. W. Sauerwein, R. Suwanarusk, B. Russell, L. Renia, F. Nosten, D. C. Tully, C. H. M. Kocken, R. J. Glynne, C. Bodenreider, D. A. Fidock, T. T. Diagana, E. A. Winzeler, Targeting *Plasmodium* PI(4)K to eliminate malaria. *Nature* **504**, 248–253 (2013). doi:10.1038/nature12782 Medline

59. E. S. Istvan, N. V. Dharia, S. E. Bopp, I. Gluzman, E. A. Winzeler, D. E. Goldberg, Validation of isoleucine utilization targets in *Plasmodium falciparum*. *Proc. Natl. Acad. Sci. U.S.A.* **108**, 1627–1632 (2011). doi:10.1073/pnas.1011560108 Medline

60. B. Baragaña, I. Hallyburton, M. C. S. Lee, N. R. Norcross, R. Grimaldi, T. D. Otto, W. R. Proto, A. M. Blagborough, S. Meister, G. Wirjanata, A. Ruecker, L. M. Upton, T. S. Abraham, M. J. Almeida, A. Pradhan, A. Porzelle, T. Luksch, M. S. Martínez, T. Luksch, J. M. Bolscher, A. Woodland, S. Norval, F. Zuccotto, J. Thomas, F. Simeons, L. Stojanovski, M. Osuna-Cabello, P. M. Brock, T. S. Churcher, K. A. Sala, S. E. Zakutansky, M. B. Jiménez-Díaz, L. M. Sanz, J. Riley, R. Basak, M. Campbell, V. M. Avery, R. W. Sauerwein, K. J. Dechering, R. Noviyanti, B. Campo, J. A. Frearson, I. Angulo-Barturen, S. Ferrer-Bazaga, F. J. Gamo, P. G. Wyatt, D. Leroy, P. Siegl, M. J. Delves, D. E. Kyle, S. Wittlin, J. Marfurt, R. N. Price, R. E. Sinden, E. A. Winzeler, S. A. Charman, L. Bebrevska, D. W. Gray, S. Campbell, A. H. Fairlamb, P. A. Willis, J. C. Rayner, D. A. Fidock, K. D. Read, I. H. Gilbert, A novel multiple-stage antimalarial agent that inhibits protein synthesis. *Nature* **522**, 315–320 (2015). doi:10.1038/nature14451 Medline

61. C. Boss, H. Aissaoui, N. Amaral, A. Bauer, S. Bazire, C. Binkert, R. Brun, C. Bürki, C. L. Ciana, O. Corminboeuf, S. Delahaye, C. Dollinger, C. Fischli, W. Fischli, A. Flock, M. C. Frantz, M. Girault, C. Grisostomi, A. Friedli, B. Heidmann, C. Hinder, G. Jacob, A. Le Bihan, S. Malrieu, S. Mamzed, A. Merot, S. Meyer, S. Peixoto, N. Petit, R. Siegrist, J. Trollux, T. Weller, S. Wittlin, Discovery and characterization of ACT-451840: An antimalarial drug with a novel mechanism of action. *ChemMedChem* **11**, 1995–2014 (2016). Medline

62. E. Sonoiki, C. L. Ng, M. C. S. Lee, D. Guo, Y.-K. Zhang, Y. Zhou, M. R. K. Alley, V. Ahyong, L. M. Sanz, M. J. Lafuente-Monasterio, C. Dong, P. G. Schupp, J. Gut, J. Legac, R. A. Cooper, F.-J. Gamo, J. DeRisi, Y. R. Freund, D. A. Fidock, P. J. Rosenthal, A potent antimalarial benzoxaborole targets a *Plasmodium falciparum* cleavage and polyadenylation specificity factor homologue. *Nat. Commun.* **8**, 14574 (2017). doi:10.1038/ncomms14574 Medline

63. G. LaMonte, M. Y.-X. Lim, M. Wree, C. Reimer, M. Nachon, V. Corey, P. Gedeck, D. Plouffe, A. Du, N. Figueroa, B. Yeung, P. Bifani, E. A. Winzeler, Mutations in the *Plasmodium falciparum* cyclic amine resistance locus (PfCARL) confer

multidrug resistance. *MBio* **7**, e00696-16 (2016). doi:10.1128/mBio.00696-16 Medline

64. C. Kimchi-Sarfaty, J. M. Oh, I.-W. Kim, Z. E. Sauna, A. M. Calcagno, S. V. Ambudkar, M. M. Gottesman, A "silent" polymorphism in the *MDR1* gene changes substrate specificity. *Science* **315**, 525–528 (2007). doi:10.1126/science.1135308 Medline

65. MalariaGEN *Plasmodium falciparum* Community Project, Genomic epidemiology of artemisinin resistant malaria. *eLife* **5**, e08714 (2016). Medline

66. B. Witkowski, V. Duru, N. Khim, L. S. Ross, B. Saintpierre, J. Beghain, S. Chy, S. Kim, S. Ke, N. Kloeung, R. Eam, C. Khean, M. Ken, K. Loch, A. Bouillon, A. Domergue, L. Ma, C. Bouchier, R. Leang, R. Huy, G. Nuel, J.-C. Barale, E. Legrand, P. Ringwald, D. A. Fidock, O. Mercereau-Puijalon, F. Ariey, D. Ménard, A surrogate marker of piperaquine-resistant *Plasmodium falciparum* malaria: A phenotype-genotype association study. *Lancet Infect. Dis.* **17**, 174–183 (2017). doi:10.1016/S1473-3099(16)30415-7 Medline

67. P. Cingolani, A. Platts, L. Wang, M. Coon, T. Nguyen, L. Wang, S. J. Land, X. Lu, D. M. Ruden, A program for annotating and predicting the effects of single nucleotide polymorphisms, SnpEff: SNPs in the genome of *Drosophila melanogaster* strain w^{1118}; iso-2; iso-3. *Fly* **6**, 80–92 (2012). doi:10.4161/fly.19695 Medline

68. M. Krzywinski, J. Schein, I. Birol, J. Connors, R. Gascoyne, D. Horsman, S. J. Jones, M. A. Marra, Circos: An information aesthetic for comparative genomics. *Genome Res.* **19**, 1639–1645 (2009). doi:10.1101/gr.092759.109 Medline

69. M. P. Glenn, S.-Y. Chang, C. Hornéy, K. Rivas, K. Yokoyama, E. E. Pusateri, S. Fletcher, C. G. Cummings, F. S. Buckner, P. R. Pendyala, D. Chakrabarti, S. M. Sebti, M. Gelb, W. C. Van Voorhis, A. D. Hamilton, Structurally simple, potent, *Plasmodium* selective farnesyltransferase inhibitors that arrest the growth of malaria parasites. *J. Med. Chem.* **49**, 5710–5727 (2006). doi:10.1021/jm060081v Medline

70. I. D. Goodyer, T. F. Taraschi, *Plasmodium falciparum*: A simple, rapid method for detecting parasite clones in microtiter plates. *Exp. Parasitol.* **86**, 158–160 (1997). doi:10.1006/expr.1997.4156 Medline

71. T. E. Wellems, L. J. Panton, I. Y. Gluzman, V. E. do Rosario, R. W. Gwadz, A. Walker-Jonah, D. J. Krogstad, Chloroquine resistance not linked to *mdr*-like genes in a *Plasmodium falciparum* cross. *Nature* **345**, 253–255 (1990). doi:10.1038/345253a0 Medline

72. S. G. Valderramos, J.-C. Valderramos, L. Musset, L. A. Purcell, O. Mercereau-Puijalon, E. Legrand, D. A. Fidock, Identification of a mutant PfCRT-mediated chloroquine tolerance phenotype in *Plasmodium falciparum*. *PLOS Pathog.* **6**, e1000887 (2010). doi:10.1371/journal.ppat.1000887 Medline

73. S. Pelleau, E. L. Moss, S. K. Dhingra, B. Volney, J. Casteras, S. J. Gabryszewski, S. K. Volkman, D. F. Wirth, E. Legrand, D. A. Fidock, D. E. Neafsey, L. Musset,

Adaptive evolution of malaria parasites in French Guiana: Reversal of chloroquine resistance by acquisition of a mutation in *pfcrt*. *Proc. Natl. Acad. Sci. U.S.A.* **112**, 11672–11677 (2015). doi:10.1073/pnas.1507142112 Medline

74. C. Kidgell, S. K. Volkman, J. Daily, J. O. Borevitz, D. Plouffe, Y. Zhou, J. R. Johnson, K. Le Roch, O. Sarr, O. Ndir, S. Mboup, S. Batalov, D. F. Wirth, E. A. Winzeler, A systematic map of genetic variation in *Plasmodium falciparum*. *PLOS Pathog.* **2**, e57 (2006). doi:10.1371/journal.ppat.0020057 Medline

75. D. S. Peterson, D. Walliker, T. E. Wellems, Evidence that a point mutation in dihydrofolate reductase-thymidylate synthase confers resistance to pyrimethamine in falciparum malaria. *Proc. Natl. Acad. Sci. U.S.A.* **85**, 9114–9118 (1988). doi:10.1073/pnas.85.23.9114 Medline

76. M. J. Manary, S. S. Singhakul, E. L. Flannery, S. E. R. Bopp, V. C. Corey, A. T. Bright, C. W. McNamara, J. R. Walker, E. A. Winzeler, Identification of pathogen genomic variants through an integrated pipeline. *BMC Bioinformatics* **15**, 63 (2014). doi:10.1186/1471-2105-15-63 Medline

77. G. A. Van der Auwera, M. O. Carneiro, C. Hartl, R. Poplin, G. Del Angel, A. Levy-Moonshine, T. Jordan, K. Shakir, D. Roazen, J. Thibault, E. Banks, K. V. Garimella, D. Altshuler, S. Gabriel, M. A. DePristo, From FastQ data to high confidence variant calls: The Genome Analysis Toolkit best practices pipeline. *Curr. Protoc. Bioinformatics* **43**, 11.10.1–11.10.33 (2013). Medline

78. A. McKenna, M. Hanna, E. Banks, A. Sivachenko, K. Cibulskis, A. Kernytsky, K. Garimella, D. Altshuler, S. Gabriel, M. Daly, M. A. DePristo, The Genome Analysis Toolkit: A MapReduce framework for analyzing next-generation DNA sequencing data. *Genome Res.* **20**, 1297–1303 (2010). doi:10.1101/gr.107524.110 Medline

79. M. A. DePristo, E. Banks, R. Poplin, K. V. Garimella, J. R. Maguire, C. Hartl, A. A. Philippakis, G. del Angel, M. A. Rivas, M. Hanna, A. McKenna, T. J. Fennell, A. M. Kernytsky, A. Y. Sivachenko, K. Cibulskis, S. B. Gabriel, D. Altshuler, M. J. Daly, A framework for variation discovery and genotyping using next-generation DNA sequencing data. *Nat. Genet.* **43**, 491–498 (2011). doi:10.1038/ng.806 Medline

80. E. Bultrini, K. Brick, S. Mukherjee, Y. Zhang, F. Silvestrini, P. Alano, E. Pizzi, Revisiting the *Plasmodium falciparum* RIFIN family: From comparative genomics to 3D-model prediction. *BMC Genomics* **10**, 445 (2009). doi:10.1186/1471-2164-10-445 Medline

81. J. Peters, E. Fowler, M. Gatton, N. Chen, A. Saul, Q. Cheng, High diversity and rapid changeover of expressed *var* genes during the acute phase of *Plasmodium falciparum* infections in human volunteers. *Proc. Natl. Acad. Sci. U.S.A.* **99**, 10689–10694 (2002). doi:10.1073/pnas.162349899 Medline

82. A. Claessens, W. L. Hamilton, M. Kekre, T. D. Otto, A. Faizullabhoy, J. C. Rayner, D. Kwiatkowski, Generation of antigenic diversity in *Plasmodium falciparum* by structured rearrangement of *Var* genes during mitosis. *PLOS Genet.* **10**, e1004812 (2014). doi:10.1371/journal.pgen.1004812 Medline

83. R Core Team, *R: A Language and Environment for Statistical Computing* (R Foundation for Statistical Computing, 2008).

84. C. Aurrecoechea, J. Brestelli, B. P. Brunk, J. Dommer, S. Fischer, B. Gajria, X. Gao, A. Gingle, G. Grant, O. S. Harb, M. Heiges, F. Innamorato, J. Iodice, J. C. Kissinger, E. Kraemer, W. Li, J. A. Miller, V. Nayak, C. Pennington, D. F. Pinney, D. S. Roos, C. Ross, C. J. Stoeckert Jr., C. Treatman, H. Wang, PlasmoDB: A functional genomic database for malaria parasites. *Nucleic Acids Res.* **37**, D539–D543 (2009). doi:10.1093/nar/gkn814 Medline

85. L. J. Nkrumah, R. A. Muhle, P. A. Moura, P. Ghosh, G. F. Hatfull, W. R. Jacobs Jr., D. A. Fidock, Efficient site-specific integration in *Plasmodium falciparum* chromosomes mediated by mycobacteriophage Bxb1 integrase. *Nat. Methods* **3**, 615–621 (2006). doi:10.1038/nmeth904 Medline

86. S. H. Adjalley, M. C. Lee, D. A. Fidock, A method for rapid genetic integration into *Plasmodium falciparum* utilizing mycobacteriophage Bxb1 integrase. *Methods Mol. Biol.* **634**, 87–100 (2010). doi:10.1007/978-1-60761-652-8_6 Medline

87. M. C. Lee, P. A. Moura, E. A. Miller, D. A. Fidock, *Plasmodium falciparum* Sec24 marks transitional ER that exports a model cargo via a diacidic motif. *Mol. Microbiol.* **68**, 1535–1546 (2008). doi:10.1111/j.1365-2958.2008.06250.x Medline

88. B. B. Czaban, A. Forer, The kinetic polarities of spindle microtubules in vivo, in crane-fly spermatocytes. I. Kinetochore microtubules that re-form after treatment with colcemid. *J. Cell Sci.* **79**, 1–37 (1985). Medline

89. M. Chanama, P. Chitnumsub, Y. Yuthavong, Subunit complementation of thymidylate synthase in *Plasmodium falciparum* bifunctional dihydrofolate reductase-thymidylate synthase. *Mol. Biochem. Parasitol.* **139**, 83–90 (2005). doi:10.1016/j.molbiopara.2004.09.010 Medline

90. O. Trott, A. J. Olson, AutoDock Vina: Improving the speed and accuracy of docking with a new scoring function, efficient optimization, and multithreading. *J. Comput. Chem.* **31**, 455–461 (2010). Medline

91. G. M. Morris, R. Huey, W. Lindstrom, M. F. Sanner, R. K. Belew, D. S. Goodsell, A. J. Olson, AutoDock4 and AutoDockTools4: Automated docking with selective receptor flexibility. *J. Comput. Chem.* **30**, 2785–2791 (2009). doi:10.1002/jcc.21256 Medline

92. The PyMOL Molecular Graphics System, Version 1.8 (Schrödinger, 2016).

93. A. J. Saldanha, Java Treeview—extensible visualization of microarray data. *Bioinformatics* **20**, 3246–3248 (2004). doi:10.1093/bioinformatics/bth349 Medline
